# Genotyping-by-sequencing-based high-resolution mapping reveals a single candidate gene for the grapevine veraison locus *Ver1*

**DOI:** 10.1093/plphys/kiae272

**Published:** 2024-05-14

**Authors:** Lena Frenzke, Franco Röckel, Torsten Wenke, Florian Schwander, Konrad Grützmann, Julia Naumann, Falk Zakrzewski, Tom Heinekamp, Maria Maglione, Anja Wenke, Anja Kögler, Eva Zyprian, Andreas Dahl, Franz Förster, Reinhard Töpfer, Stefan Wanke

**Affiliations:** Institute of Botany, Technische Universität Dresden, 01062 Dresden, Germany; Julius Kühn Institute (JKI), Institute for Grapevine Breeding Geilweilerhof, 76833 Siebeldingen, Germany; asgen GmbH & Co . KG, 01069 Dresden, Germany; Julius Kühn Institute (JKI), Institute for Grapevine Breeding Geilweilerhof, 76833 Siebeldingen, Germany; asgen GmbH & Co . KG, 01069 Dresden, Germany; Institute of Botany, Technische Universität Dresden, 01062 Dresden, Germany; asgen GmbH & Co . KG, 01069 Dresden, Germany; Julius Kühn Institute (JKI), Institute for Grapevine Breeding Geilweilerhof, 76833 Siebeldingen, Germany; Julius Kühn Institute (JKI), Institute for Grapevine Breeding Geilweilerhof, 76833 Siebeldingen, Germany; Institute of Botany, Technische Universität Dresden, 01062 Dresden, Germany; Institute of Botany, Technische Universität Dresden, 01062 Dresden, Germany; Julius Kühn Institute (JKI), Institute for Grapevine Breeding Geilweilerhof, 76833 Siebeldingen, Germany; DRESDEN-concept Genome Center, Center for Molecular and Cellular Bioengineering, Technische Universität Dresden, 01307 Dresden, Germany; Institute of Botany, Technische Universität Dresden, 01062 Dresden, Germany; Julius Kühn Institute (JKI), Institute for Grapevine Breeding Geilweilerhof, 76833 Siebeldingen, Germany; Institute of Botany, Technische Universität Dresden, 01062 Dresden, Germany; Departamento de Botánica, Instituto de Biología, Universidad Nacional Autónoma de México, 04510 Mexico City, Mexico; Botanik und Molekulare Evolutionsforschung, Senckenberg Forschungsinstitut und Naturmuseum, 60325 Frankfurt am Main, Germany; Institut für Ökologie, Evolution und Diversität, Goethe-Universität, 60438 Frankfurt am Main, Germany

## Abstract

Veraison marks the transition from berry growth to berry ripening and is a crucial phenological stage in grapevine (*Vitis vinifera*): the berries become soft and begin to accumulate sugars, aromatic substances, and, in red cultivars, anthocyanins for pigmentation, while the organic acid levels begin to decrease. These changes determine the potential quality of wine. However, rising global temperatures lead to earlier flowering and ripening, which strongly influence wine quality. Here, we combined genotyping-by-sequencing with a bioinformatics pipeline on ∼150 F_1_ genotypes derived from a cross between the early ripening variety “Calardis Musqué” and the late-ripening variety “Villard Blanc”. Starting from 20,410 haplotype-based markers, we generated a high-density genetic map and performed a quantitative trait locus analysis based on phenotypic datasets evaluated over 20 yrs. Through locus-specific marker enrichment and recombinant screening of ∼1,000 additional genotypes, we refined the originally postulated 5-mb veraison locus, *Ver1*, on chromosome 16 to only 112 kb, allowing us to pinpoint the ethylene response factor *VviERF027* (VCost.v3 gene ID: Vitvi16g00942, CRIBIv1 gene ID: VIT_16s0100g00400) as veraison candidate gene. Furthermore, the early veraison allele could be traced back to a clonal “Pinot” variant first mentioned in the seventeenth century. “Pinot Precoce Noir” passed this allele over “Madeleine Royale” to the maternal grandparent “Bacchus Weiss” and, ultimately, to the maternal parent “Calardis Musqué”. Our findings are crucial for ripening time control, thereby improving wine quality, and for breeding grapevines adjusted to climate change scenarios that have a major impact on agro-ecosystems in altering crop plant phenology.

## Teaser

Genetic mapping reveals a single candidate gene controlling the onset of ripening in grapevine.

## Introduction

The quality and character of a wine result from the interplay between grapevine (*Vitis vinifera* L.) cultivars and local growing conditions. However, climate change is altering local growing conditions. Indeed, rising temperatures and increasingly frequent heat waves (Dosio et al. 2018; IPCC 2023) influence grapevine phenology, as higher temperatures over the last decade have affected the onset and duration of the grapevine growth cycle (Venios et al. 2020). These changes cause a shift in vine phenological stages depending on the cultivar and location and negatively affect berry composition, altering traditional wine styles and varietal characteristics (Droulia and Charalampopoulos 2022; Ramos and Yuste 2023).

Producing high-quality wine in the face of current and predicted future environmental conditions will require short- and long-term responses. In the short term, growers must carefully select specific varieties for a specific location; in the long term, breeders must have a vision for variety breeding to produce robust plants adapted to climate change. This vision must take into account key traits such as the transition from berry growth to berry ripening, known as veraison. Veraison is a crucial developmental stage that is marked by changes such as berry softening, the onset of sugar accumulation, and degradation of organic acids (Barnavon et al. 2000). In addition, the biosynthesis of anthocyanin compounds in red grape varieties changes the color of berries from green to red, while berries of white grape varieties turn yellowish and lose their brightness (Zyprian et al. 2018).

The introduction of marker-assisted selection (MAS) has increased the efficiency of grapevine breeding programs, paving the way from the classical empirical cross design to a predictive approach based on the molecular and genetic characterization of the grapevine. Resource optimization during breeding is achieved by selecting parental plants with favorable alleles as well as early removal of undesirable genotypes (Töpfer et al. 2011; Delrot et al. 2020). MAS is particularly important for perennial crop breeding, where the period from planting to first fruit set takes years, usually 3 yrs in grapevine. The subsequent in-depth evaluation during breeding, in particular to assess the quality potential of breeding lines, currently requires at least two decades.

The population derived from a cross between the early ripening variety “Calardis Musqué” and the mid- to late-ripening variety “Villard Blanc” (CM × VB) is a perfect resource for studying veraison, as this phenotype clearly segregates in the progeny. Zyprian et al. (2016) discovered a major quantitative trait locus (QTL) for veraison, namely *Ver1*, in this population and mapped it to chromosome 16. Flanking markers and confidence intervals led to the localization to a ∼5-mb region of the reference genome sequence. The *Ver1*-linked simple sequence repeat (SSR) marker UDV-052 was tested and reported as a potential screening marker for early veraison in different cultivars by Zyprian et al. (2018) and included in meta-QTL analyses by Delfino et al. (2019). SSRs have been commonly used in grapevine breeding research over the last two decades (e.g. Schwander et al. 2012; Fechter et al. 2014; Rex et al. 2014; Pap et al. 2016; Migliaro et al. 2019). These markers remain useful due to their robustness, reproducibility, multiallelic information content, and direct transferability to cost-efficient and reliable SSR-based MAS pipelines. However, SSRs are of limited use when developing genome-wide high-resolution genetic maps, which may hamper extensive fine-mapping of multiple traits and identifying candidate genes. Now, high-throughput sequencing methods (Bennett et al. 2005; Margulies et al. 2005), along with large-scale parallelization and automation, can produce vast quantities of data points, such as single-nucleotide polymorphisms (SNPs) at low cost. Genotyping-by-sequencing (GBS) allows for SNP discovery and specific genetic fingerprinting at the same time. GBS enables the reproducible detection of loci at the same genomic position even at low coverage and thus with reduced costs (Elshire et al. 2011). Since Barba et al. (2014) began using GBS of an F_1_ grapevine population to map resistance loci, GBS-based SNP data have been widely applied for genetic mapping of *Vitis* varieties (e.g. Hyma et al. 2015; Teh et al. 2017; Sapkota et al. 2019; Tello et al. 2019; Fu et al. 2020; Possamai et al. 2021). However, due to their inherent biallelic nature and low information content, the usefulness of these resulting individual SNPs is limited for genetic mapping approaches (Buntjer et al. 2005). These limitations can be overcome by considering adjacent SNPs as haplotype-based markers (HBMs), which are then generally multiallelic and simplify allelic phasing (Bhat et al. 2021).

Here, we developed a bioinformatics pipeline to extract informative HBMs from GBS data and constructed a high-density integrated genetic map from a segregating population of grapevine (*Vitis vinifera* L.). This map and veraison data evaluated over 20 yrs were used for QTL analysis. Applying locus-specific marker enrichment (LSME) and recombinant screening of ∼1,000 additional genotypes lead to a marked narrowing of the *Ver1* locus interval on chromosome 16 to only 112 kb. This, supported by published evidence, allows the pinpointing of the veraison candidate gene, the ethylene response factor (ERF) *VviERF027* (VCost.v3 gene ID: Vitvi16g00942). Furthermore, the early veraison allele was traced back to a clonal “Pinot” variant with one of the earliest mentions in 1,766 (Sprenger 1766). The genetic map and approaches presented in this study will facilitate the discovery and development of wine quality trait-related markers relevant for breeding grapevine varieties adapted to future climates.

## Results

### *In silico* digestion and repeat content exploration of the grapevine genome

Our GBS analysis was designed as a low-coverage approach that minimizes sequencing costs without limiting marker density. We performed an *in silico* digestion of the PN40024 12× reference genome sequence, which returned between 31,789 and 840,898 fragments depending on the restriction enzyme tested (Supplementary Fig. S1A). *Psi*I (restriction site TTA^TAA) yielded 423,837 fragments, of which 120,257 fragments ranged between 200 and 700 bp, a suitable size for short-read high-throughput sequencing approaches. These fragments will produce about 240,000 reads from paired-end sequencing. Thus, 30× to 60× coverage per restriction site should be expected for the haploid genome (see Materials and methods section on “Genotyping-by-sequencing strategy design”). Given that grapevine is diploid, these numbers are equivalent to the coverage per allele at a heterozygous locus. Hence, we chose *Psi*I as the restriction enzyme to digest genomic DNA prior to library preparation from all genotypes. A RepeatExplorer analysis indicated that about 70% of the grapevine genome is composed of repetitive sequences, with the remaining 30% representing the unique genome fraction (Supplementary Fig. S1B). The number of expected unique loci from the original 120,257 restriction fragments should therefore be around 36,000 at a ratio of 70:30.

### Determination of loci and allelic segregation

**Figure 1.**
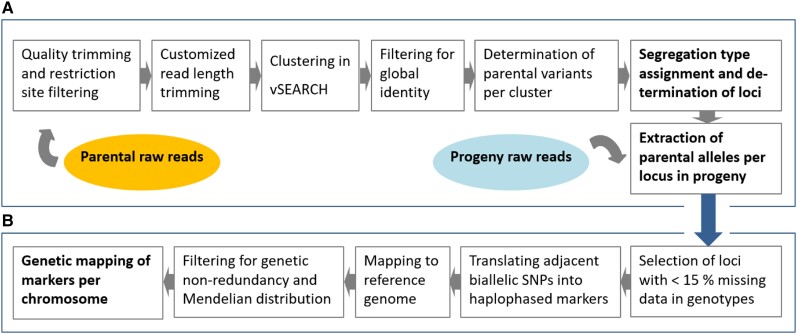
Diagram of the bioinformatics workflow to obtain genetic markers for subsequent QTL analysis. **A)** Filtering and clustering of parental raw reads obtained from GBS data to assign segregation types and to obtain allelic information for loci in the progeny. **B)** Additional selection steps for markers and subsequent correlation analyses to obtain an integrated genetic map.

We applied a bioinformatics workflow that minimizes missing data per genotype, thus making optimal use of the sequence data information while also being economical in terms of data processing (see Materials and methods for details). Accordingly, we analyzed the *Psi*I loci and corresponding alleles on a read-length level rather than on a de novo or reference-based assembly. The individual steps included filtering and clustering of parental GBS raw reads, assigning segregation types and progeny allelic information (Fig. 1A). Further marker selecting steps and subsequent correlation and QTL analyses resulted in an integrated genetic map (Fig. 1B).

We analyzed 15 million parental TAA-reads and 2 million TAA-reads (*Psi*I-cutting site) per F_1_ individual. In this dataset, we detected 561,795 SNPs in comparison with the reference genome, most of which were monomorphic (67%, Supplementary Fig. S2) or biallelic (21%) (Table 1). About 7.3% of all SNPs represented three-allelic loci, with another 2.2% for four-allelic loci. The bi, three-, and four-allelic loci were, on average, defined by a set of three, five, or seven adjacent SNPs, respectively. The pipeline we implemented also allowed us to identify insertions and deletions (InDels). In total, we identified 64,104 InDels (Supplementary Fig. S3).

**Table 1.** Distribution of loci, alleles, and HBMs in the F_1_ mapping population

Locus type	No. of alleles	No. of loci	No. of uniquely located loci on the reference genome (19 chrs)	HBMs (≥ 85% data completeness)	Used for genetic mapping	Applicable for LSME
Homozygous/monomorphic	1	342,270	94,707	NA	0%	0%
Heterozygous/polymorphic	2	105,282	49,410	4,793 *hk* × *hk* 3,662 *nn* × *np* 4,680 *lm* × *ll*	0%	64%
	3	36,940	21,446	4,811 *ef* × *eg* 484 *nn* × *np* 855 *lm* × *ll*	30%	30%
	4	11,265	8,070	1,125 *ab* × *cd*	6%	6%
	**Total**	**495,757**	**173,633**	**20,410**	**36%**	**100%**

Markers segregating as ab × cd and ef × eg are fully informative.

NA, not applicable; LSME, locus-specific marker enrichment.

**Figure 2.**
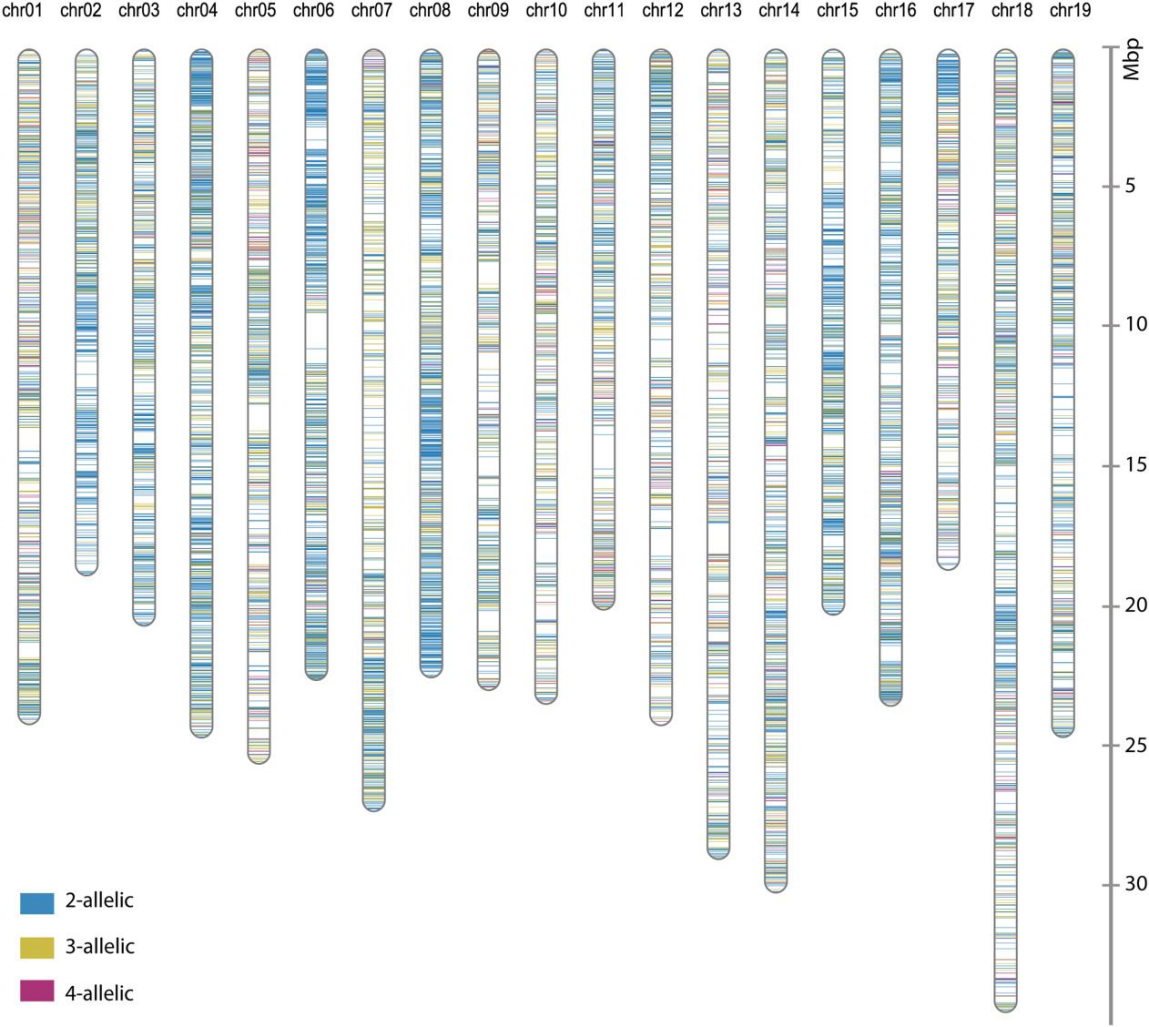
Physical mapping of the HBMs reveals high coverage of *Vitis vinifera* chromosomes. Physical map of 19,440 HBMs with informative segregation that were shared by ≥85% of the F_1_ progeny. Individual distributions of the bi-, three−, and four-allelic loci along the reference chromosomes are provided as Supplementary Figs. S4, S5, and S6, respectively. Mbp, mega base pairs; chr, chromosome.

**Figure 3.**
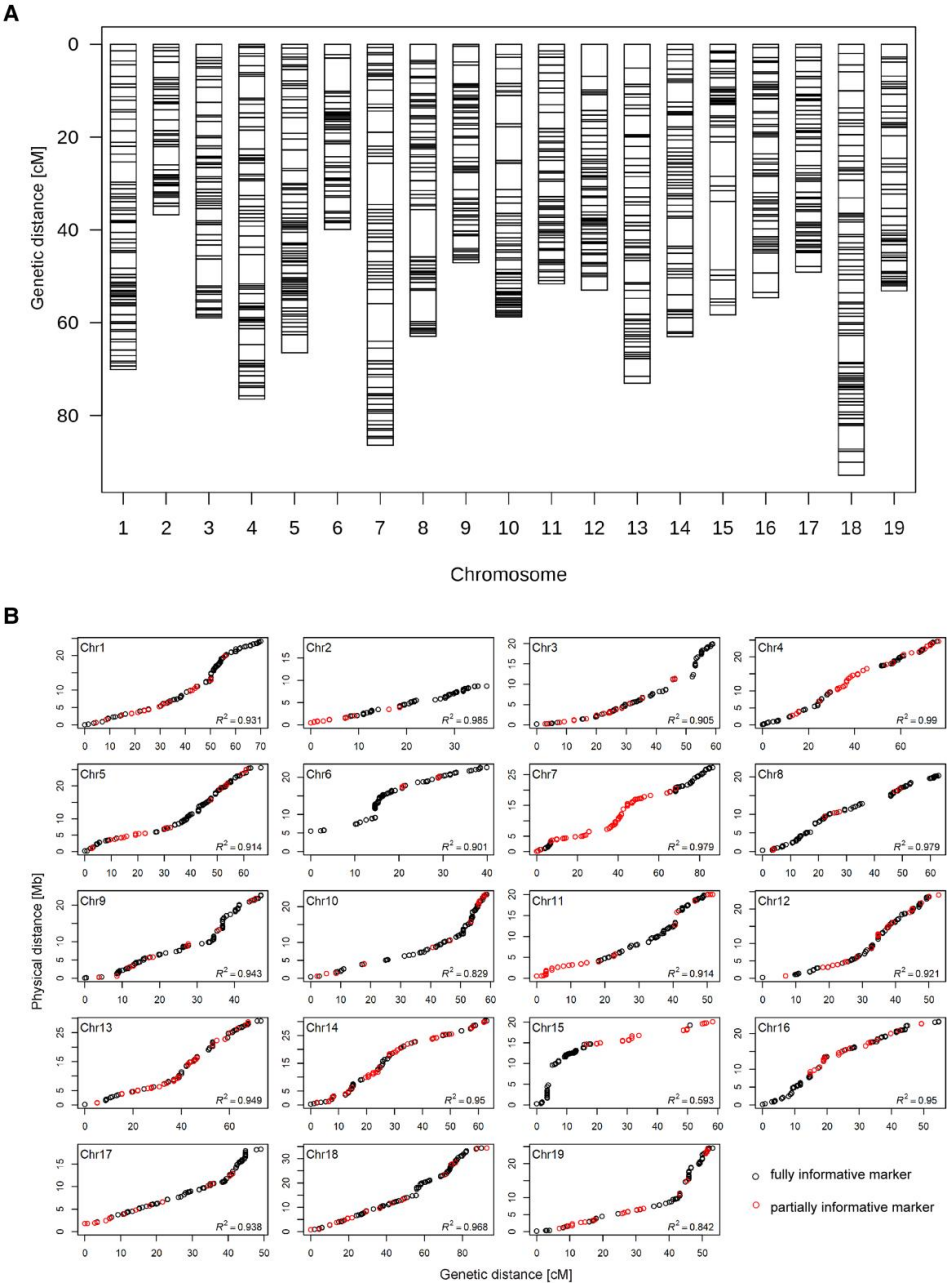
Integrated genetic map and Marey maps based on HBMs. **A)** Integrated genetic map. **B)** Marey maps showing the coincidence between physical and genetic positions for the analyzed F_1_ population. Chromosome numbers and their orientation are based on the reference genome PN12×v2 (Canaguier et al. 2017).

After filtering the loci data for ≥85% completeness across all progeny, we assigned the segregation types to the extracted 20,410 loci (Table 1). We processed the resulting ∼6,000 HBMs, representing fully informative loci, for genetic mapping. We also used the remaining ∼13,500 HBMs based on partially informative loci together with the fully informative HBMs for LSME. Of the 20,410 HBMs, we successfully mapped 19,440 (or 95.2%) by BLAST to the 19 chromosomes of the *V. vinifera* reference genome (haploid, 486 mb; Canaguier et al. 2017) (Fig. 2, Supplementary Figs. S4 to S6). We assigned the HBMs to chromosomes based on the BLAST hits against the reference genome to avoid having interspersed markers without a known position. We limited the final number of HBMs per linkage group (LG) to 110 to 125 to reduce the computational effort for QTL analysis. We selected the most suitable markers per physical 1-Mb fragment in order to achieve a largely even distribution of markers along the chromosomes (see Materials and methods for details). The final integrated genetic map contained 2,260 HBMs covering an average genetic distance of 60.65 cM per chromosome and 1,152.29 cM in total (Fig. 3A; Supplementary Table S1). Of these HBMs, 1,826 (∼80%) were fully informative with segregation patterns *ab* × *cd* or *ef* × *eg*. The revised SSR map (see Materials and methods for details) consisted of 394 SSR markers defining 19 LGs with an average inter-marker distance of 4.1 cM and a total length of 1,621 cM (Supplementary Fig. S7; Supplementary Table S1). Of the 394 SSR markers, 63% showed fully informative segregation patterns. Compared to this revised SSR map, the mean number of markers per chromosome in the GBS-based map increased from about 20 to 119 and the mean inter-marker distance decreased from 4.1 to 0.51 cM (Supplementary Table S1). The average *R*^2^ of the collinearity between the genetic and physical distance increased from 0.83 to 0.92 (Fig. 3B and Supplementary Fig. S8).

### QTL analysis for onset of veraison

**Figure 4.**
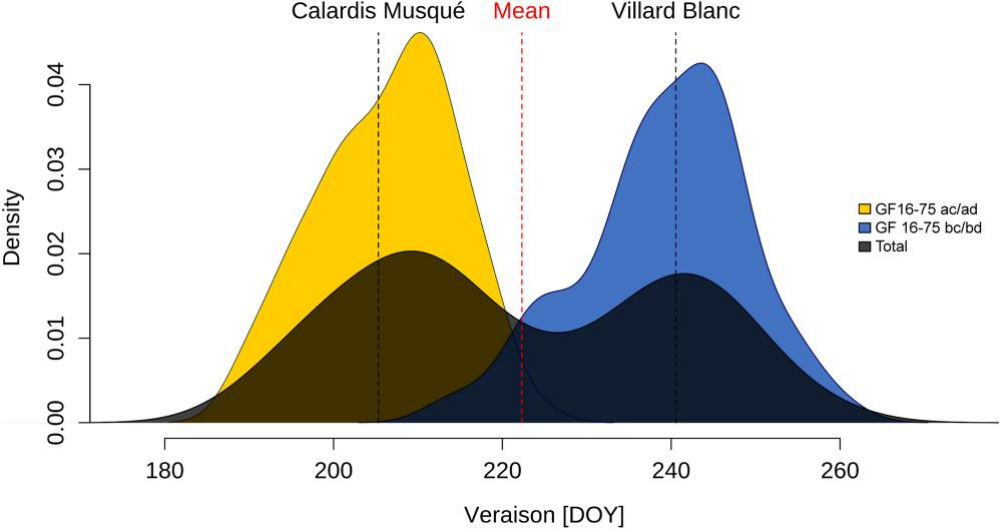
Distribution of veraison time across the F_1_ population. BLUP-adjusted mean density plot of onset of veraison for the entire population and separated according to the individual marker segregation for the main QTL on chromosome 16 at SSR marker GF16-75. Total mean and parent-specific means for the population are indicated by dashed lines. DOY—day of the year.

Three main phenological stages determine the seasonal developmental cycle of grapevines: budburst, flowering, and veraison. The veraison dates for the F_1_ individuals were determined and recorded in 17 individual datasets over 22 yrs in three different field plots (FPs). The distribution of best linear unbiased prediction (BLUP)-adjusted mean veraison date over all years exhibited two peaks (Fig. 4, black area). The first maximum was on day of the year (DOY) 210 (corresponding to July 29) and the second on DOY 243 (August 31), aligning well with the veraison date of the respective parents “Calardis Musqué” and “Villard Blanc”. All pairwise correlations between individual datasets were particularly high and significant (*P*-value < 0.001) (Supplementary Fig. S9). Furthermore, the genomic heritability estimates for all single datasets based on the SSR and GBS maps ranged from 0.66 in 1999 to 0.99 in 2016, demonstrating strong genetic influence and reliability independently of annual fluctuations in environmental factors (Supplementary Fig. S10).

A first QTL analysis was performed regarding onset of veraison using this population by Zyprian et al. (2016) with an SSR- and SNP-based genetic map based on five phenotypic datasets. To compare these initial results with those obtained with our high-density GBS-based map with all 17 datasets, we revised the initial genetic map and repeated the QTL analysis with the enhanced phenological dataset of 17 seasons using identical parameters. We confirmed the main QTL for veraison on chromosome 16 in all 17 datasets and for their BLUP (Table 2; Supplementary Table S2). When performing interval mapping (IM), the maximum logarithm of the odds (LOD_max_) based on the SSR map ranged from 11.9 to 30.9, while it varied from 12.3 to 30.1 when using the GBS map for all individual datasets acquired between 1998 and 2020 (Table 2). The phenotypic variance explained by this QTL for the individual datasets ranged from 43% to 62%. In general, composite interval mapping (CIM) confirmed the presence of a major locus on chromosome 16 with LOD_max_ ranging from 15.1 to 60.5 and explaining 46.9% to 81.1% of phenotypic variance for all individual datasets summarized for both maps (Table 2; Supplementary Table S2). We also detected minor QTLs, barely exceeding the genome-wide logarithm of odds (LOD) threshold, on chromosomes 2, 7, 13, 17, and 18 (Table 2; Supplementary Table S2). We observed the minor QTL on chromosome 17 in 8 of 17 datasets, with a LOD score ranging from 5.4 to 9.5 and from 4.5 to 6.8 for the SSR and GBS maps, respectively. This QTL was not detected by Zyprian et al. (2016). Based on IM analysis, this QTL explained roughly 8% to 14.5% of the phenotypic variance. Since we observed this in multiple datasets using both maps and mapping methods, we propose to name this locus *Ver3*. Based on the IM results, *Ver3* cosegregates with the marker UDV-092 (position in the reference genome: 9,613,080 bp) on the SSR map and with marker L82652 (position in the reference genome: 9,701,912 bp) on the GBS map.

**Table 2.** Summary of QTL analysis for veraison onset

	SSR map				GBS map			
	IM		CIM		IM		CIM	
LG	Datasets with QTL	LOD (range)	Datasets with QTL	LOD (range)	Datasets with QTL	LOD (range)	Datasets with QTL	LOD (range)
2	7 +BLUP	2.5 to 3.1	1	5.4	7 +BLUP	2.7 to 3.4	−	−
7	11 +BLUP	2.9 to 6.7	−	−	10 +BLUP	3.2 to 6.3	−	−
13	9	2.6 to 3.7	1	5.3	6	2.9 to 3.6	−	−
16	17 +BLUP	11.9 to 30.9	17 +BLUP	16.9 to 60.5	17 +BLUP	12.3 to 30.1	17 +BLUP	15.1 to 50.1
17	9 +BLUP	2.9 to 5.0	8	5.4 to 9.5	8 +BLUP	2.7 to 4.8	8 +BLUP	4.5 to 6.8
18	6	2.9 to 3.6	1	5.8	7 +BLUP	2.9 to 3.9	1	4.8

Data are shown for QTLs present in at least six out of 17 individual datasets, separated based on the map and analysis methods used.

IM, interval mapping; CIM, composite interval mapping; BLUP, best linear unbiased predictors; LOD, logarithm of the odds; LG, linkage group.

**Figure 5.**
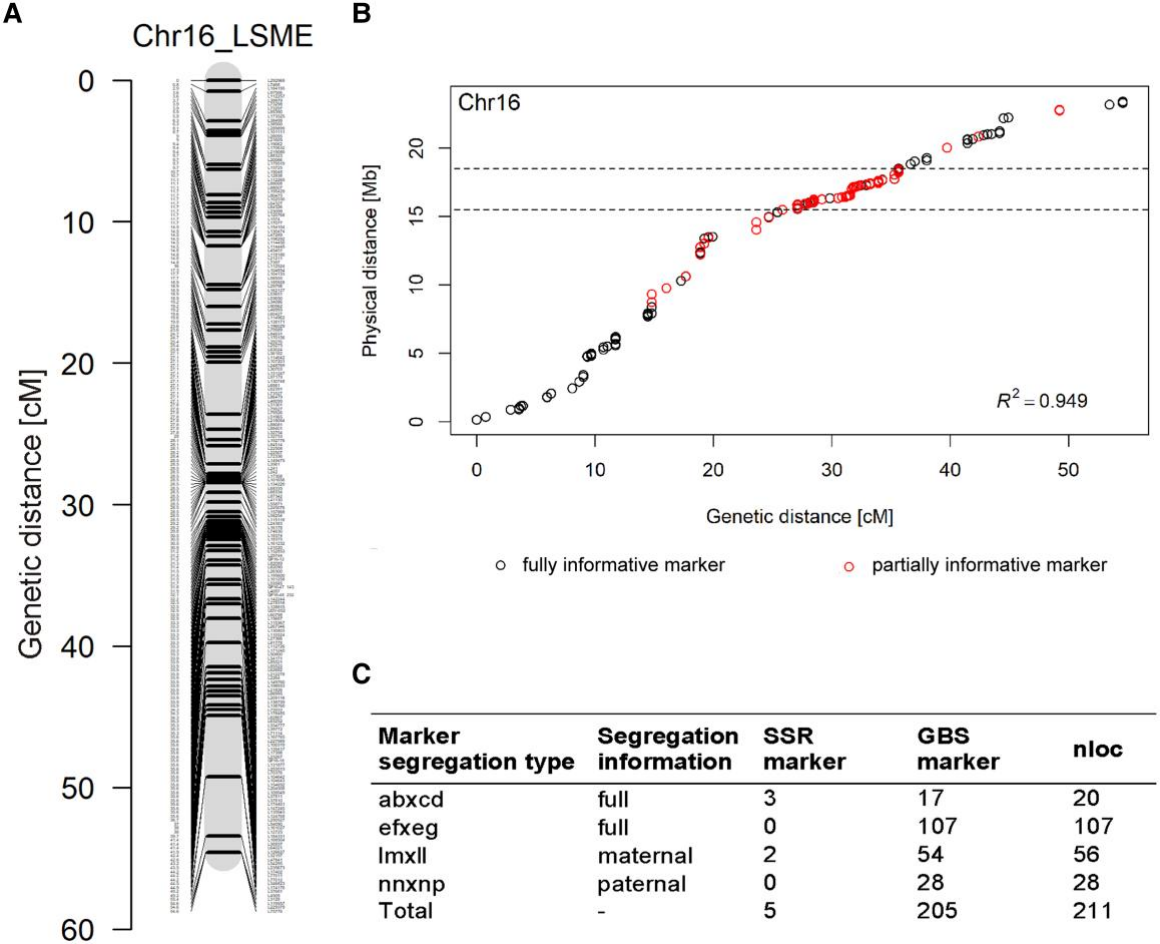
Integrated LSME map to fine-map the major veraison QTL *Ver1* on chromosome 16. **A)** Integrated genetic map for chromosome 16 (physical position 15.5 to 18.5 mb) based on SSR and GBS markers. For details on marker names and positions, see Supplementary Table S3. **B)** Marey map showing the coincidence between physical and genetic positions of the LSME map. **C)** Marker segregation types of the integrated LSME map. Chr, chromosome; nloc, number of loci.

**Figure 6.**
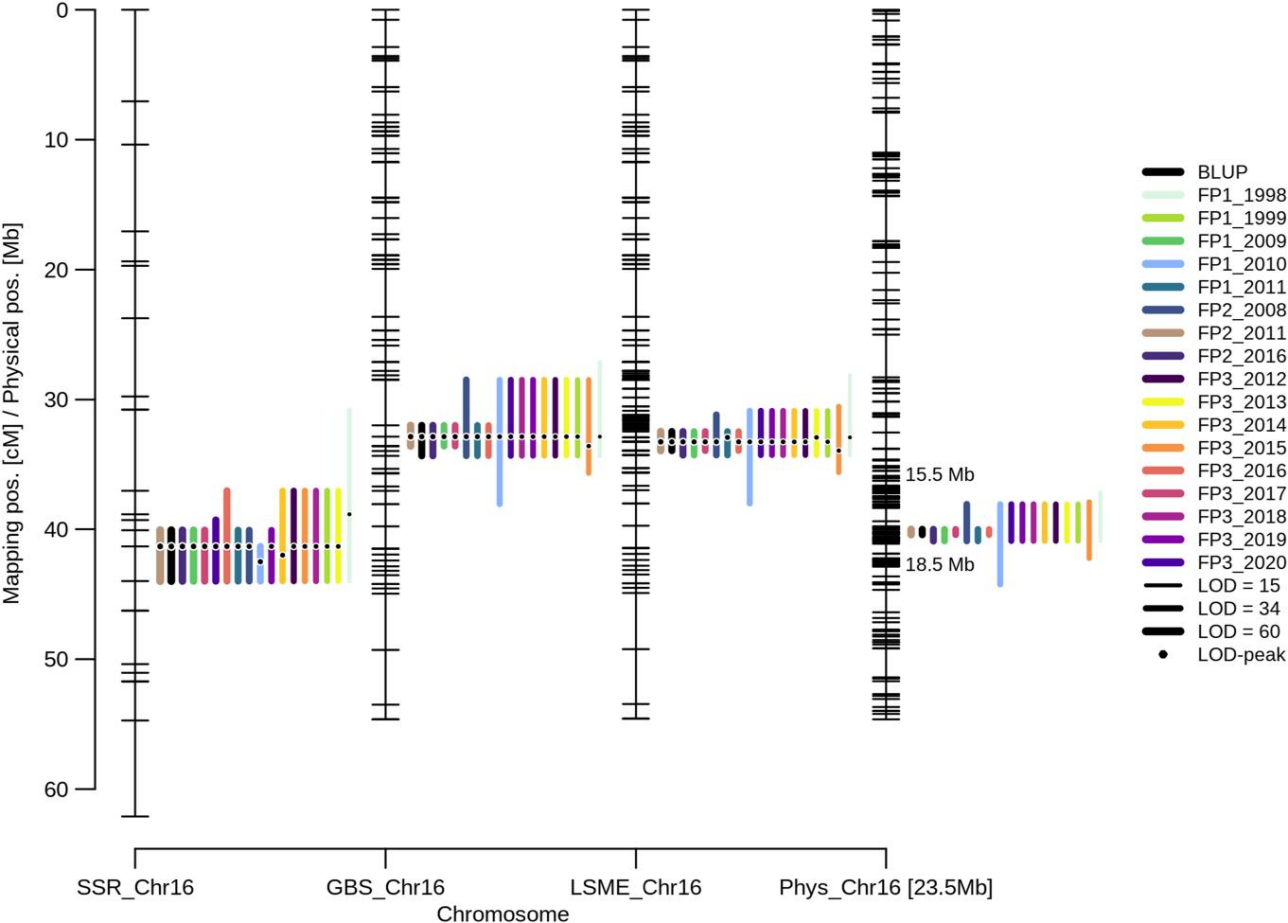
Fine-mapping of the veraison locus *Ver1*. CIM logarithm-of-odds (LOD_max_-2) confidence intervals of the major veraison QTL *Ver1* on chromosome 16 for all analyzed years and resulting multi-year and multi-plot BLUP for SSR, GBS, and LSME map-based approaches. The physical map of chromosome 16 is given with all mapped markers and the relative LSME results scaled to the total map size of the LSME_Chr16 map. Line thickness of the intervals indicates minimum, mean, and maximum LOD as indicated in the legend. SSR, simple sequence repeat; GBS, genotyping-by-sequencing; Chr, chromosome; FP, field plot; BLUP, best linear unbiased predictor.

To narrow down the mapping intervals of each QTL, we developed an R pipeline, LSME, which we applied to the major locus *Ver1* on chromosome 16 for the physical interval from 15.5 to 18.5 mb. The algorithm assessed a combined list containing 19,351 markers from the SSR and GBS approach, of which about 6,000 were fully informative (Supplementary Fig. S11). We constructed a local, high-density genetic map using these markers, integrated it in the original map (Fig. 5, Supplementary Table S3), and repeated the CIM analysis (Fig. 6; Supplementary Table S2). From this approach, we narrowed down the location of the *Ver1* locus using the veraison BLUP to a 178-kb interval between the markers L128815 and L34171 (positions in the reference genome: 17,262,831 bp and 17,441,186 bp). For all single years, the genotypes with the allelic combinations “*ac*” and “*ad*” at SSR marker UDV-052 with the highest LOD value in the SSR map showed earlier veraison onset compared to the “*bc*” and “*bd*” genotypes. This result is exemplified for the BLUP of veraison onset across all years in Supplementary Fig. S12. From this analysis, we conclude that early veraison onset was inherited from the maternal parent “Calardis Musqué” and originated from the maternal grandparent “Bacchus Weiss”. In addition, the value from the genotypic class “*ac/ad*” was significantly different from that of the class “*bc/bd*” within each individual year, with large Cohen's *d* effect sizes spanning from 1.92 in 1998 to 4.88 in 2011 (Supplementary Table S4).

#### Validation by recombinant analysis

**Figure 7.**
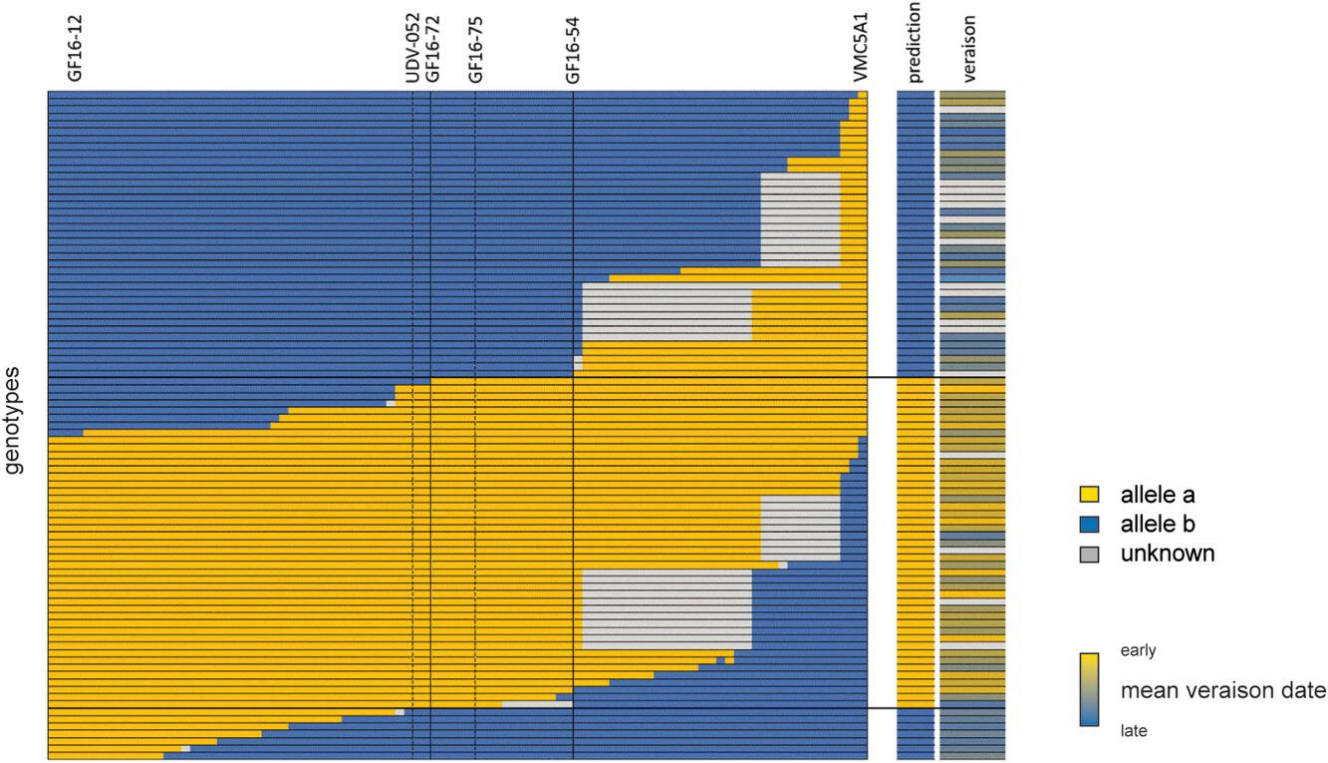
Delimiting the *Ver1* locus. The location of *Ver1* was based on 91 genotypes from the CM × VB population with recombination events in a 6.4-Mb region covered by 68 HBMs and 21 SSR markers surrounding the *Ver1* locus on chromosome 16. Recombination events delimit the *Ver1* locus to a 112-kb region between markers GF16-72 and GF16-54. Allele *a* from the “Bacchus Weiss” allele of “Calardis Musqué” is linked to early veraison. Outer right column shows color-coded mean veraison dates of genotypes. For details, see Supplementary Table S5.

Twelve of the original 147 mapped F_1_ genotypes showed recombination events within a large 6.4-Mb *Ver1* interval (15.0 to 21.4 mb; flanked by markers GF16-12 and VMC5A1, Supplementary Table S5). We screened an additional 995 offspring of the CM × VB cross with SSR markers on chromosome 16, yielding 79 individuals with potential recombination events within the *Ver1* region. We subjected these accessions to genotyping with 68 locus-specific HBMs and 21 locus-specific SSR markers (Fig. 7). We scored the veraison date for these additional recombinants for the 2021, 2022, and 2023 seasons, from which we calculated a mean DOY for veraison for all individuals. As these veraison data are based on single grapevine plants with their own root systems (and not grafted onto other rootstocks), veraison date is strongly influenced by the vigor, vitality, and yield status of the individual vine, which leads to a less precise dataset compared to the long-term BLUP values of the mapping population. Nevertheless, the data allowed the delineation of the *Ver1* locus to between GF16-72 (17,322,483 bp) and GF16-54 (17,434,058 bp), resulting in a final *Ver1* interval of 112 kb (Supplementary Table S5). The identified central marker GF16-75 offers a slightly improved accuracy and MAS applicability compared to marker UDV-052 applied by Zyprian et al. 2018 (Fig. 7, prediction column). As expected, the observed veraison dates of the recombinants grouped according to the GF16-75 allele pattern differed significantly (Supplementary Fig. S13).

## Discussion

### Relevance of veraison

Due to climate change, new breeding strategies are urgently needed to enable sustainable viticulture. The time of ripening is a fundamental criterion for the usability of a grapevine cultivar in a specific growing region. This phenomenon was realized early on and quantified by the Huglin index (Huglin 1986), whereby cultivars are attached to specific temperature zones within wine-growing regions. Whereas bud break and flowering represent the beginning of the annual vegetative and reproductive growth periods, respectively, veraison marks the onset of berry ripening, with major changes in berries related to wine quality (Conde et al. 2007; Delfino et al. 2019). Thus, veraison is an efficient target to adjust ripening behavior in breeding to modulate the quality potential of a variety under given climatic conditions. Notably, with noticeable warming worldwide due to climate change, traditional grapevine cultivars tend to ripen too early, especially in traditional wine-growing regions with cooler climates (Koch and Oehl 2018). This early ripening can result in a compressed harvest period, leading to processing-related problems and concerns for winemakers. Recently, Gomès et al. (2021) stated that a variety should be able to maintain a good acidity and aroma level, even under high temperatures. Sprightliness and aromatic potential of white wines cultured in cool climates largely depend on an optimal “ripening window” (warm during the day, cool nights). High night temperatures during ripening can lead to a rapid degradation of malic acid and adversely affect aroma formation, resulting in unbalanced wines with low acidity and high alcohol content. Veraison should therefore not be too early to avoid high alcohol content and low acidity in the wine, but also not too late to avoid processing unripe grapes and thus insufficient aroma expression in the wine. Cultivars with predicted suitable ripening periods thus represent a crucial breeding goal to maintain unique wine quality features (Duchêne 2016; Töpfer and Trapp 2022).

### Climate change-adapted breeding material

In breeding programs, trait-linked genetic markers are the basis for MAS to assist with phenotypic selection. GBS is an application of high-throughput sequencing for discovering SNPs as well as size-limited InDels that can be used for marker development. A crucial step in making MAS more practical is the validation of trait-linked markers for diverse breeding material, since a marker present in an analyzed population may be absent from another genotype. Within the 112-kb interval of the *Ver1* locus on LG 16, we detected 15 InDels and 46 SNPs from the GBS data (Supplementary Fig. S14). These InDels and SNPs will be the first targets for marker development for retracing haplophases associated with early or late veraison in further breeding material. Additionally, we tested SSR markers flanking the putative *Ver1* locus that can directly be applied to the long-established SSR-based MAS pipelines of many breeding institutions. We propose GF16-75 as an alternative SSR marker given that it is more tightly linked to veraison than marker UDV-052. In breeding programs with respective crosses, negative selection can be conducted by discarding the 50% of seedlings that have inherited the “early” haplophase from “Bacchus Weiss” [or another verified “Pinot Precoce Noir” (PPN) offspring]. Such a negative selection would result in a later onset of ripening in the remaining genotypes by ∼33 days on average. Conversely, positive selection for early veraison will allow selecting genotypes for climatic areas where viticulture was previously impossible.

### ERF027 and veraison

Twelve genes are listed in the VCost.v3 annotation (Table 3; Canaguier et al. 2017) for the 112-kb physical interval of the *Ver1* locus. Zyprian et al. (2016) proposed three transcription factor genes within the previously established ∼5-mb mapping interval: Vitvi16g00941 (CRIBIv1 gene ID: VIT_16s0100g00380), Vitvi16g01860 (CRIBIv1 gene ID: VIT_16s0100g00390), and Vitvi16g00942 (hereinafter referred to as *VviERF027*; CRIBIv1 gene ID: VIT_16s0100g00400), by analyzing the genes closely linked to the maximum LOD SSR marker UDV-052 (start position 17,267,916 bp). The best candidate gene may be the ethylene-responsive transcription factor gene Vitvi16g00942 with the highest similarity to *ETHYLENE RESPONSE FACTOR 027 (ERF027)* from Arabidopsis (*Arabidopsis thaliana*). Fasoli et al. (2018) created a detailed transcriptomic and metabolomic map of berry development for the cultivars “Pinot Noir” (PN) and “Cabernet Sauvignon” and listed *VviERF027* as the only gene from the fine-mapped 112-kb interval as a putative biomarker gene for veraison onset, based on its expression profile showing a peak at veraison and a decline thereafter. Furthermore, Theine et al. (2021) analyzed transcriptomic changes during berry development of PN and its early ripening mutant “PPN”. The expression of *VviERF027* started around 2 wk earlier in PPN than in PN, while showing equally high expression, as determined by transcriptome deep sequencing (RNA-seq) and confirmed by reverse-transcription quantitative PCR. The temporal shift coincides with the difference of 2 wk in veraison onset between the two cultivars. The authors proposed *VviRTIC1* (VIT_210s0071g01145) and *VviRTIC2* (VIT_200s0366g00020) as possibly being responsible for the phenotypic differences between these two cultivars. In our QTL study, we observe the phenotypic effect of the inherited early ripening PPN allele compared to its absence (Fig. 4). This together with the equally high expression in both PN and PPN could be due to the earlier veraison known for PN compared to most international varieties [PN classified as “early” (note 3) according to the International Organisation of Vine and Wine (OIV) code 303; OIV Descriptor List https://www.oiv.int/node/3028]. The differential expression for *VviERF027* has also been reported in the early ripening table grape mutant “Summer Black” (Wei et al. 2020) and during fruit development (Fang et al. 2023). Palumbo et al. (2014) already identified *VviERF027* as one of 190 grapevine switch genes in the berry transcriptome that were expressed at a low level during the immature phase and were then significantly induced at veraison in a set of five red-berry grapevine cultivars.

**Table 3.** Candidate genes within the mapping interval of *Ver1* on chromosome 16

VCost.v3 gene ID	CRIBIv1 gene ID	Start (bp)	End (bp)	BLASTx RefSeq prediction
Vitvi16g00938	VIT_16s0100g00370	17,274,084	17,324,348	Valine-tRNA ligase, mitochondrial 1 isoform X1
Vitvi16g00941	VIT_16s0100g00380	17,325,124	17,325,780	Dehydration-responsive element-binding protein 1B isoform X1
Vitvi16g01860	VIT_16s0100g00390	17,330,110	17,343,918	Ethylene-responsive transcription factor ERF025
Vitvi16g00942	VIT_16s0100g00400	17,348,815	17,349,893	Ethylene-responsive transcription factor ERF027
Vitvi16g00943	–	17,350,062	17,350,486	–
Vitvi16g00944	VIT_16s0100g00410	17,359,769	17,366,257	traB domain-containing protein isoform X1
Vitvi16g00946	VIT_16s0100g00420	17,368,383	17,373,765	Two-component response regulator ARR2
Vitvi16g00947	VIT_16s0100g00430	17,380,424	17,385,531	Two-component response regulator ARR1
Vitvi16g00948	–	17,394,333	17,399,723	Two-component response regulator ARR14-like
Vitvi16g01446	VIT_16s0100g00440	17,405,415	17,406,086	LOB domain-containing protein 24
Vitvi16g00949	VIT_16s0100g00450	17,413,804	17,419,307	Serine/arginine-rich splicing factor RS41 isoform X1
Vitvi16g00951	VIT_16s0100g00460	17,430,838	17,432,693	Protein DETOXIFICATION 49

Grapevine reference genome annotations with gene IDs are reviewed in Grimplet and Cramer (2019).

Transcription factors of the APETALA2/ERF (AP2/ERF) family are one of the predominant transcription factor families in plants and play a crucial role in fruit ripening (as reviewed in Zhai et al. 2022). The regulation is mediated by the AP2 binding domain and members of the ERF subfamily specifically bind to the so-called GCC-box (containing the AGCCGCC element, Hao et al. 1998). This element is often found in upstream regions of genes that respond to pathogens, ethylene, and wounding (Ohme-Takagi and Shinshi 1995). We identified this typical ERF-binding element within the 2,000-bp putative promoter region in 21 of the 388 veraison-specific differentially expressed genes (DEGs) coexpressed with our candidate gene *VviERF027* (at 41, 49, and 56 days after flowering in Theine et al. 2021; Supplementary Table S6. The expanded OneGeneNetwork (Pilati et al. 2021; Pearson’s corr. 0.6 to 1.0, OneGeneThreshold 0.97 to 1.0) of our candidate gene contains 25 genes (Supplementary Fig. S15). Within this network, a group of 16 genes are closely linked to each other through multi-cascade interactions. Ten of them belong to the 388 co-expressed veraison-specific DEGs (Theine et al. 2021), including Vitvi02g01019 (*VviMYBA1*) and Vitvi13g00035 (VIT_13s0067g00700), which have the GCC-Box as potential binding site in their upstream-flanking region (Supplementary Fig. S15). Identification of *VviMYBA1* is to be expected in cultivars with red-skinned berries (as investigated in PN and PPN in Theine et al. 2021), as there is high evidence that this gene triggers anthocyanin biosynthesis to induce the most obvious signal of veraison in red cultivars (Matus et al. 2017). All genotypes of our cross population have white berry color, and since the promoter region of *VviMYBA1* is nonfunctional due to a *Gret1* retrotransposon insertion in white cultivars (Walker et al. 2007), a differential expression is not expected within the given genotypes. However, this result underscores the identification of *VviERF027* as an upstream gene in the genetic pathway that initiates maturation. The second putative GCC-Box-associated target gene is Vitvi13g00035 (VIT_13s0067g00700), which is described as potential alpha/beta hydrolase-related protein (MapMan at2g40095). This gene was found to have a 4-fold increased expression in naphthalene acetic acid-treated berries with delayed ripening of Merlot (Ziliotto et al. 2012), highlighting its potential role in the signaling cascade initiated by *VviERF027*. A suspected involvement of *VviERF027* in repressing pathways of maturation is indicated by the co-expressed DEGs that negatively correlate to our candidate gene in the gene network (Supplementary Fig. S15, red lines), such as VIT_15s0046g00290 (auxin response factor) and VIT_19s0085g00010 (auxin-responsive protein *SAUR71*). However, the delay of ripening onset by auxin (Böttcher et al. 2011, 2013) and the absence of a GCC motif in the promoter region of both genes might only suggest a downstream interaction of our candidate gene in this process. It is also noteworthy that the gene network shows a link between *VviERF27* and the directly neighboring VIT_16s0100g00390, which is also located within the QTL region, but according to Fasoli et al. (2018) and Theine et al. (2021) has no identified expression.

In addition, *VviERF027* showed an expression profile consistent with the accumulation of monoterpenes in Moscato Bianco (VIVC-No. 8193; prime name: Muscat à petits grains blancs) ripening berries and was listed as one of the most significant candidate genes for monoterpene biosynthesis by Costantini et al. (2017). This is not surprising, as it has long been known that the formation of monoterpenes starts with veraison and that monoterpene concentration increases with advancing maturity (Gunata et al. 1985). This suggests the possible diverse involvement of our candidate gene in an even more complex regulatory network, although we found no overlap with the extracted group of DEGs from Theine et al. (2021).

It is known that active transposable elements (TEs) can influence the transcription of neighboring genes (reviewed in Slotkin and Martienssen 2007). If a genome contains many copies of a repeat family of DNA transposons or retrotransposons with very high identity, i.e. if only few mutations have accumulated over time, these TEs may still be active. We searched for corresponding TEs in the *VviERF027* region (30-kb upstream and 30-kb downstream) using local BLASTn within the grapevine reference genome (Canaguier et al. 2017) and performed alignments and remappings of hits. Although this region is characterized by the presence of many repeats of different families, only highly divergent or incomplete copies were identified, which can hardly be considered potentially active, and thus should not affect the expression of *VviERF027*.

Both the results of our fine-mapping approach and comparisons with previous expression studies indicate that *VviERF027* is most likely the gene causing the ∼33-day variance in maturation time within the respective population. The analysis of promoter motifs and gene network correlations allow only limited speculation about the exact regulatory role of *VviERF027*, which deserves validation by functional approaches.

#### Origin of the early veraison allele

The allelic combinations “*ac*” and “*ad*” resulted in earlier veraison onset across all study years. Thus, early veraison onset was inherited from the maternal parent “Calardis Musqué” and originated from the maternal grandparent “Bacchus Weiss”. “Bacchus Weiss” is a direct descendant of the early ripening “Mueller-Thurgau Weiss” according to VIVC (https://www.vivc.de/), which most likely inherited the trait from “Madeleine Royale” (pedigree tree: https://www.vivc.de/index.php? r=passport/viewtree&id=851). “Madeleine Royale” is the result of a cross between a member of the “Pinot'-clone family and “Schiava Grossa”. Since “Madeleine Royale” is early ripening and “Schiava Grossa” is not, we conclude that the true second parent of “Madeleine Royale” may be “PPN”, which inherited the early ripening trait. Guo et al. (2019a, 2019b) analyzed the early ripening trait in descendants of the cultivar “Pearl of Csaba” (Prime name: “Csaba Gyoengye’), a grandchild of “Madeleine Royal”, and were able to associate early ripening inheritance with marker VVMD5 located in proximity to *Ver1* on chromosome 16. More recently, a resequencing approach on the “Pearl of Csaba” pedigree identified conserved haplotypes and candidate genes for early ripening in more detail. A gene encoding a folate/biopterin transporter (*VviFBT*) with a missense mutation and mapping to the upper part of chromosome 16 (VIT_16s0039g00720) was identified to be specifically and highly expressed during grapevine berry development, particularly at veraison (He et al. 2023). In summary, based on inheritance tracking of the phenotype, the early veraison trait, and possibly the *Ver1* locus, may have originated from “PPN” and was passed over four generations to “Calardis Musqué”, the maternal parent of the analyzed population, causing the variation in veraison observed in the F_1_ population analyzed in this study.

### Optimized data mining procedures for genetic mapping

We designed our GBS pipeline to maximize the gain of information from DNA sequencing data. Therefore, we minimized early filtering steps at different levels of the approach, resulting in a large dataset of ∼500,000 loci. Most GBS approaches in *Vitis* use *ApeK*I, a methylation-sensitive restriction enzyme, to a priori exclude repetitive regions from genotyping (Hyma et al. 2015; Tello et al. 2019; Sapkota et al. 2019; Supplementary Table S7). Instead, we used a restriction enzyme that is not sensitive to methylation (*Psi*I) to expand the number of potential markers by including loci from repetitive regions showing at least 90% identity in our customized GBS approach. Heterozygous under-calling in shallow sequencing approaches like GBS is a major problem, especially in the case of highly heterozygous and diverse species like grapevine (Ward et al. 2013; Swarts et al. 2014). We thus deeply sequenced the parental genotypes to identify reliable heterozygous loci in low-coverage data of the progeny. To improve the number of initial loci, our computational pipeline is based on de novo clustering and alignment on the read-length level. With this procedure, we were able to identify not only SNPs but also InDels that might represent valuable candidates for marker development followed by application using selective PCR, as shown for the grapevine downy mildew resistance locus *Rpv3-1* (Foria et al. 2018). Importantly, our modular de novo approach is taxon independent, does not necessarily rely on a reference genome, and allows to incorporate different marker types (SNPs, SSRs, InDels). Consequently, it is highly versatile and expandable to future application platforms.

A comparison of our genotyping and mapping approach to a selection of recently published studies on bi-parental populations or subpopulations is compiled in Supplementary Table S7. These studies vary widely regarding population design and size, genotyping approach, bioinformatics pipeline, and marker terminology, making direct comparisons challenging. Although the amount of characterized HBMs uncovered in the present study (20,410) is within the range of previous studies (1,351 to 65,229), our GBS approach identified a unique level of information content among markers. Fully informative markers made up 1.2% of our initial loci set and 29% of the HBMs characterized. By contrast, the proportion of fully informative markers in other studies ranges from 0.004% to 1% of the initial loci set and only 0.09% to 12% of HBMs (Supplementary Table S7; Zhu et al. 2018; Sapkota et al. 2019; Tello et al. 2019; Fu et al. 2020; Su et al. 2020). Our GBS map covers 1,152.29 cM, thus showing an average inter-marker distance of 0.51 cM. These values lie at the lower end of the range that has been reported for *Vitis* genetic maps (860.46 to 3,014.46 cM, 0.05 to 3.7 cM) (Supplementary Table S7).

### Optimized local marker enrichment

A locus for the beginning of ripening on chromosome 16 was first described by Fischer et al. (2004) in a “Regent’ × “Blaufränkisch” population (called LG R1 in Fischer et al. 2004). Although the available positional information is not always comparable, further associations to LG 16 were described by Costantini et al. (2008), Duchêne et al. (2012), and Guo et al. (2019a). Furthermore, Delfino et al. (2019) performed a meta-QTL analysis for the veraison trait and suggested two subregions on chromosome 16 covering about 3.3 mb and 173 annotated genes. We used long-term veraison data to validate the major QTL *Ver1* on LG 16 (Zyprian et al. 2016) and narrowed down its location to a 178-kb interval by LSME (Fig. 6; Supplementary Table S2) and to a 112-kb interval by recombinant analysis. *Ver1* represents a highly significant QTL present in all analyzed years that explained up to 81% of the phenotypic variance.

## Materials and methods

### Plant materials

The segregating F_1_ mapping population was created in 1989 at the Julius Kühn Institute (JKI), Institute for Grapevine Breeding, Geilweilerhof, Germany, by crossing the white-berried and fungus-resistant grapevine (*Vitis vinifera*) cultivars “Calardis Musqué” (VIVC-No. 4549; synonym GF.GA-47-42; “Bacchus Weiss” × “‘Seyval”) and “Villard Blanc” (VIVC-No. 13081; “Seibel 6468”' × ‘“Subereux”). The progeny consist of 150 unselected genotypes cultivated in three directly adjacent FPs at Geilweilerhof (N49°21.675, E8°04.433). In field plot 1 (FP1), the original seedlings were planted with their own root systems in 1996. Additional clones of the population were planted with their own root systems with two plants per genotype in 2000 (field plot 2, FP2) as described by Zyprian et al. 2016. Field plot 3 (FP3) was established in 2010 and consists of eight clonally propagated vines per genotype grafted onto the rootstock “Selektion Oppenheim 4’ (VIVC-No. 11473) with a vertical shoot positioning trellis system (10 to 12 buds) and a plant density of 5,000 vines per hectare (2 × 1 m spacing). Parental plants were co-cultivated in the same plots. A set of 995 genotypes, crossed in 2013 from the same parents and planted in a separate FP at Geilweilerhof with their own root systems in 2014, was used to identify additional recombinants.

### Phenotyping for veraison

The parental individuals show distinct ripening behaviors. “Calardis Musqué” is known for its early ripening, whereas “Villard Blanc” is mid- to late ripening. Plants from the parents and individuals from the population were evaluated for date of veraison (trait_id CO_356:2000002), which was converted to DOY for QTL analysis. For the present study, previously published data were integrated for five growing seasons: 1998, 1999, 2008, 2009, and 2010. The onset of veraison was determined by testing ten representative berries per cluster on at least three clusters per plant. Veraison was defined as the stage in which 15% to 20% of the fruits softened (manually assessed) and turned bright green (Zyprian et al. 2016). In the 2011 to 2016 seasons, veraison rating was adapted to the OIV descriptor 303. The date of veraison was recorded when 50% of the berries in representative bunches were classified as soft by manual inspection. Veraison data from 2017 to 2020 were interpolated from Fourier transform infrared spectroscopy measurements (WineScan SO2 Auto, Foss, Reilingen, Germany) throughout the (pre-)ripening period and determined when a density value of 1.025 was measured using GrapeScan calibration (Integrator Software, Foss, Reilingen, Germany) adapted for each year based on the results of a round robin test between German wine analysis laboratories. The suitability of the interpolation under the given conditions is validated by clear correlation between veraison datasets (Supplementary Fig. S9). For this study, 17 individual phenotypic veraison datasets (FP1: 1998, 1999, 2009, 2010, 2011; FP2: 2008, 2011, 2016; FP3: 2012 to 2020) were included. The variance in datasets due to different rating methods and seasons was considered and removed by calculating a BLUP value over all seasons and plots. For phenotyping of the additionally identified recombinant genotypes in the years 2021, 2022, and 2023, the OIV-adapted method was used.

### Statistical analysis

For statistical analysis of veraison datasets, the R software environment (https://www.r-project.org/) was used. BLUPs (Piepho et al. 2008) for all phenotyped years were determined with the R-package *phenotype* (Zhao 2020), and genomic heritability (*h^2^*) estimates were calculated for each individual dataset using the R-package *heritability* (Kruijer et al. 2015). Spearman’s rank correlation coefficients with corresponding *P* values were computed with the R core package *stats*. Before QTL analysis, in order to achieve a normal distribution, individual veraison datasets were Box-Cox power transformed with the R-package *AID* (Dag et al. 2017). Allelic effects of the maximum LOD marker were computed with the R-package *ggstatsplot* (Patil 2021), and the effect sizes of Cohen's d were computed with the R-package *lsr* (Navarro 2022).

### DNA extraction

For DNA extraction, three to four young leaves per genotype were freshly collected on ice and frozen or immediately freeze-dried upon arrival in the laboratory. High molecular–weight genomic DNA was extracted from 20 mg of dried leaf material using a modified hexadecyltrimethylammonium bromide (CTAB) protocol (Doyle and Doyle 1990) with CTAB (4% w/v). The final DNA concentration and quality were assessed using a Qubit dsDNA HS Assay Kit (Thermo Fisher Scientific) and a genomic DNA 50KB Kit (#DNF-467, Agilent) on a Fragment Analyzer (Agilent Technologies Inc.). For further details on the extraction protocol, see Supplementary Information S1.

### GBS strategy design

To estimate the number of fragments and sequencing depth required for GBS, a comparative *in silico* restriction analysis was performed, as well as a RepeatExplorer analysis. The target amount of next-generation sequencing (NGS) reads to be generated for each genotype was estimated to be around 15 million. Suitable restriction enzymes were determined by a virtual digestion applying the insilico.digest function of the R package SimRAD (v0.96; Lepais and Weir 2014) to the latest grapevine reference assembly (haploid, 486 mb, Canaguier et al. 2017; https://urgi.versailles.inra.fr/Species/Vitis/Data-Sequences/Genome-sequences). Twenty-five restriction enzymes were tested with a 6-bp recognition motif and are not sensitive to methylation (Supplementary Fig. S1A). For genotyping and later QTL analyses, we focused on the unique genome fraction (that is, excluding repetitive sequences). Information on the repeat fraction of the genome is therefore crucial to estimate a priori the potential number of useful restriction fragments. Accordingly, 4,145,400 Illumina raw reads obtained from a HiSeq 1000 instrument (raw reads deposited at European Nucleotide Archive (ENA) under the accession number SRR863595, https://www.ebi.ac.uk/ena/browser/view/SRR863595) were fed into the RepeatExplorer clustering pipeline as described in Novák et al. (2013) to analyze the repeat content of the grapevine genome prior to GBS (Supplementary Fig. S1B).

### GBS library preparation and high-throughput sequencing

Three µg of extracted genomic DNA was digested with 30 U of restriction enzyme (*Psi*I-v2, see *in silico* digestion results, Supplementary Fig. S1A) (New England BioLabs GmbH) according to the manufacturer's instructions. The digested DNA was then purified with 1× AmpliClean magnetic beads (Nimagen) and dissolved in 40 *µ*L HPLC-grade water (AppliChem GmbH). One microgram of *Psi*I-digested DNA was subjected to standard Illumina paired-end DNA library preparation, which included end polishing, A-tailing, and ligation of 15 *µ*M indexed adapters (IDT UDI Dual TruSeq Adapter, Illumina). The libraries were purified twice with 0.8× AMpure XP-beads (Beckman Coulter), eluted in 20 *µ*L of elution buffer (10 mm Tris–HCl, pH 8.0 to 8.5), and then quantified with a Qubit dsDNA HS Assay Kit. Illumina libraries were subjected to PCR amplification with input of 10 ng of ligated DNA for 15 cycles using a KAPA HyperPlus Kit (#7962428001, Roche). Final libraries were purified using 0.8× AMPure XP-beads and then quantified via a Fragment Analyzer (#DNF-474, Agilent). Up to 96 equimolar-adjusted NGS libraries were pooled and sequenced using either an Illumina NextSeq 500 or HiSeq 2500 platform at the DRESDEN-concept Genome Center of TU Dresden, generating paired 150-bp reads.

### GBS data analysis pipeline

All sequencing data were processed on a Linux server with 128 CPU and 1 TB RAM following panel A of the flowchart shown in Fig. 1. Paired-end reads from each sequencing run were quality-trimmed using trimmomatic (v0.39; Bolger et al. 2014) with the following parameters: PE ILLUMINACLIP:adapters/TruSeq3-PE.fa:2:30:10:2 LEADING:30 TRAILING:30 SLIDINGWINDOW:4:30 MINLEN:36. All forward and reverse reads, paired and unpaired, were filtered for starting with the restriction motif TAA and merged into one fastq file using the ShortRead R package (v1.44.3) (Morgan et al. 2009). Depending on the ratio of read number and length after the trimming and filtering steps, it is necessary to define the appropriate amount of data that will be further processed. Low quality at the 3′ end of the reads forcibly leads to shortened sequences or a low number of full-length reads. Therefore, the reads were further trimmed to 100 nucleotides as a conservative approach that allows us to work with minimal to no missing data per genotype (trimmomatic parameters: SE CROP:100 MINLEN:100). Parental reads were merged with the zcat command in Linux into one file (Fig. 1A). Clean parental reads were clustered with VSEARCH (v1.14, Rognes et al. 2016) using the major parameters “90% identity” and a global pairwise alignment. The VSEARCH algorithm determines a seed sequence for each cluster and aligns all reads to a corresponding cluster that is at least 90% similar to the seed sequence. However, sequences often comply with the 90% parameter to the seed but do not fit in the global context of the entire cluster. The subsequent filtering step thus removes reads of a cluster that do not match this parameter. Specific parameters were: –cluster_fast –strand both –iddef 4 –id 0.90 –uc. Resulting clusters were processed with a custom R script (available as jupyter notebook workflow-part2-process-vsearch-output.r.ipynb, Supplementary information S2). Read sequences were then merged to corresponding cluster entries via read IDs. Clusters consisting of only one sequence and sequences that only appeared once were removed. Reverse-complement sequences were inverted. All sequences within each cluster were aligned using MUSCLE (Edgar 2022). Overhanging nucleotides at the 3′ and 5′ ends were removed. Clusters with a global distance between all sequences >10% were discarded, and sequences were unicalized, keeping one read per potential allele (Fig. 1A). The resulting clusters were considered as putative genetic loci of the parents with unique sequences of parental alleles. Raw read sequences of all genotypes, including parental genotypes, were mapped to the alleles requiring exact sequence matches. At least three matching reads were defined as a present allele, <3 as an absent allele. As such, parental segregation types (for instance *ab* × *cd* and *ef* × *eg*) and allelic genotypes of offspring were determined. The segregation types within the progeny were assigned by extracting the locus-specific allelic variants from about two million reads per genotype. Loci that had plausible allele presence in fewer than 85% of offspring genotypes were discarded as likely not correctly resolved. The resulting loci and their variants were mapped against the reference genome (PN40024_12×v2, Canaguier et al. 2017) to physically anchor them. To improve the mapping sensitivity, the first three bases (TTA) of the *Psi*I restriction site (TTATAA) were added to the 100-nt sequences prior to mapping in Geneious (v11.0.3). In order to extract the genomic coordinates, the resulting SAM files were converted to BED files using the convert2bed function (BEDOPS v. 2.4.40; Neph et al. 2012). Visualization of the locus density and coordinates on the reference chromosomes was conducted using the R-package *Rideogram* (Hao et al. 2020).

### Construction of genetic maps

After filtering as described above, a genetic map was constructed using the R-package *onemap* designed for outcrossing plant species (Fig. 1B; Margarido et al. 2007). HBMs were coded according to the R package manual and filtered for genetic nonredundancy within the population with the functions *find_bins* and *create_data_bins*. In a second step, segregation distortion was calculated and only markers with a suitable Mendelian distribution (χ^2^ < 65) were retained. For mapping, markers were grouped based on their linkage, but only kept if all markers within a LG mapped to the same chromosome. This step ensures that the final map aligns with the reference genome as best as possible and strengthens the reliability of downstream candidate gene analysis. To achieve an even distribution of markers across the genome, the most suitable markers in terms of data completeness and segregation distortion (deviation from expected Mendelian ratio using Pearson’s χ^2^ test) per 1 Mb of physical length were selected, resulting in 110 to 125 markers per chromosome. The mapping to the 19 grapevine chromosomes was conducted with a reference genome-guided marker start order and the Kosambi function for calculating genetic distances. In a final step, single occurrences of double crossover events were corrected and missing data points imputed, when flanking markers showed identical segregation.

The SSR marker-based genetic map used for comparison with the GBS map is a revised version of the one published by Zyprian et al. (2016). All SSR markers were reanalyzed to remove gaps and uncertainties in the Zyprian et al. (2016) dataset. The maximum likelihood mapping option of JoinMap 4.1 software (Kyazma B.V., Wageningen, The Netherlands) was used with default settings for grouping and map construction.

### QTL analysis and LSME

IM was conducted using the R package *R/qtl* (Broman et al. 2003). Therefore, the GBS map initially constructed with the R package *onemap* (Margarido et al. 2007) was imported as a *four-way* cross (*ab* × *cd*) with known phases by recoding the marker data according to the manual. The SSR map calculated in *JoinMap* (Kyazma B.V., Wageningen, The Netherlands) was imported directly as implemented in the package. The multiple imputation method with the one-dimensional scan function *scanone* was used, and simulated genotypes were drawn with a step size of 1 cM using the function *sim.geno*. For both maps, 512 simulation replicates were performed. LOD significance levels for putative QTLs were determined with 1,000 permutations chromosome-specific and genome-wide.

CIM for both genetic maps was conducted with the R-package *fullsibQTL* specialized for outcrossing species (Gazaffi et al. 2020). Since the package uses multipoint genetic maps estimated with *onemap* (Margarido et al. 2007) as input, the GBS map was used directly. The marker data of the SSR map were recoded and the genetic map re-estimated with the same marker order for further use. For the analysis, the step size was set to 0.5 cM, and cofactors automatically selected (function: *cof_selection*) with an effect threshold of 0.001 and LOD significance were determined with 1,000 permutations on a genome-wide level.

The LSME method was developed as a fine-mapping pipeline that uses the position information of a previously detected QTL to construct a high-density local genetic map with *onemap* and repeats the CIM analysis with *fullsibQTL*. The basis was a marker dataset combining the SSR markers, the GBS markers previously used for genetic mapping, and the remaining GBS-based markers exhibiting partially informative segregation (maternal: *lm* × *ll*, paternal: *nn* × *np*, or split: *hk* × *hk*). The inputs used were the respective chromosome and the physical position of the markers flanking the QTL interval based on the reference genome. In the first step, the algorithm extracted all genetic markers from the region of interest and enforced a mapping based on the reference genome marker order. Markers that did not fit the genetic map were removed, but genetically redundant markers, i.e. those with exactly the same pattern of alleles, were retained to preserve all possible positional information in the final map. In a next step, the local genetic map was integrated into the original GBS map and CIM analysis was repeated with the same parameters to fine-map the respective locus of interest.

Confidence intervals for IM, CIM, and LSME-CIM analyses were determined with the function *lodint* integrated in the *qtl* package by expanding positions to the next nonsimulated marker (“expand to markers = true”). The chromosome plot with QTL results was drawn based on a custom version of the *segmentsonMap* function integrated in the *qtlTools* package (Lovell 2021).

### Targeted recombination analysis

A total of 995 offspring of the CM × VB (cross from 2013) F_1_ population were screened with SSR markers on chromosome 16 to identify genotypes with potential recombination events in the *Ver1* locus region. Seventy-nine accessions showed a recombination event on the maternal allele between the markers GF16-12 (16,472,526 bp) and GF16-28 (20,207,041 bp). Twenty-five of these genotypes were subsequently genotyped by applying the GBS pipeline and with 68 locus-specific HBMs. Sixteen locus-specific SSR markers were developed using WebSat (Martins et al. 2009) (Supplementary Table S5). Gaps resulting from missing data (in italics, light coloration in Supplementary Table S5) were closed when the genetic state was supported by flanking markers to develop a simplified recombination scheme.

### Candidate genes

Gene IDs and sequences of candidate genes were extracted from the 12×v2 assembly of the PN40024 grapevine reference genome (Canaguier et al. 2017) between the coordinates of the LOD_max_ ±2 confidence interval of the LSME QTL results using the veraison BLUP calculated over all individual datasets. Additionally, annotated open reading frames were aligned to the nonredundant protein sequence database RefSeq (Pruitt et al. 2007) with the local alignment tool BLASTx (Altschul et al. 1997). Based on the best hits, short descriptions of putative gene functions were extracted.

### Accession numbers

Genes we refer to in this article are listed in Table 3 and Supplementary Table S6. Their sequences can be accessed via the EMBL data library *Ensembl Plants*.

## Supplementary Material

kiae272_Supplementary_Data

## Data Availability

Demultiplexed and adapter-trimmed sequencing data were deposited in the European Nucleotide Archive (ENA) (BioProject PRJEB75005, study ERP159605). Computational notebooks and R scripts used in this paper are deposited on GitLab (https://gitlab.mn.tu-dresden.de/s2390007/GBS-data-analysis-pipeline) and archived on Zenodo (https://doi.org/10.5281/zenodo.11102599).

## Supplementary data

Supplementary Fig. S1. *In silico* tests (A) and genotyping-by-sequencing strategy design (B).

Supplementary Fig. S2. Physical map of identified monomorphic loci mapped to the reference genome PN40024_12x v2 (Canaguier et al. 2017).

Supplementary Fig. S3. Physical map of identified insertions/deletions (InDels) mapped to the reference genome PN40024_12×v2 (Canaguier et al. 2017).

Supplementary Fig. S4. Physical map of all loci with two possible alleles (2-allelic) that are shared by 85% of the F_1_ progeny.

Supplementary Fig. S5. Physical map of all loci with three possible alleles (3-allelic) that are shared by 85% of the F_1_ progeny.

Supplementary Fig. S6. Physical map of all loci with four possible alleles (4-allelic) that are shared by 85% of the F_1_ progeny.

Supplementary Fig. S7. Integrated genetic map based on SSR markers of the F_1_ population from the ‘Calardis Musqué’ × ‘Villard blanc’ cross.

Supplementary Fig. S8. Marey maps showing the coincidence between physical and genetic positions of the SSR map.

Supplementary Fig. S9. Spearman correlation matrix between single veraison datasets.

Supplementary Fig. S10. Genomic heritability estimates (*h^2^*) of the veraison trait in single-year datasets using genomic relationships between individuals for each genetic map.

Supplementary Fig. S11. Distribution of segregation types in the LSME approach for the 19,351 markers.

Supplementary Fig. S12. Allelic effects on the Best Linear Unbiased Prediction (BLUP) of the veraison onset according to the genotypic classes “*ac*” (light green), “*ad*” (light orange), “*bc*” (light purple) and “*bd*” (light pink) at the marker UDV-052 showing the highest LOD value.

Supplementary Fig. S13. Distribution of the mean veraison date of *Ver1* recombinant genotypes grouped by the genomic state of correlated marker GF16-75 with significant differentiation between maternal alleles *a* (light green, light orange) and *b* (light purple, light pink).

Supplementary Fig. S14. Chromosome 16 with the 112-kb veraison locus (*Ver1*, orange box, above). Zoomed in region (below) shows the distribution of SNPs and InDels as well as positions of major candidate genes in the corresponding region (blue).

Supplementary Fig. S15. OneGenE networks of our candidate gene *VviERF027* (Vitvi16g00942). (A) Expanded gene network with Vitvi16g00942 as seed gene in the center (yellow star). (B) Reduced OneGenE network of Vitvi16g00942 showing only genes shared with the 388 DEGs of Theine et al. (2021).

Supplementary Table S1. Marker saturation of linkage groups for the integrated SSR and GBS map.

Supplementary Table S2. Total QTL (quantitative trait locus) analysis results.

Supplementary Table S3. SSR and GBS markers of the 15.5 to 18.5 mb region on chromosome 16 based on integrated locus-specific marker enrichment (LSME).

Supplementary Table S4. Two-sample *t*-test for genotypic classes *ac/ad* against *bc/bd* at marker UDV-052 showing the highest LOD value.

Supplementary Table S5. Recombinant genotypes from the CM × VB population with recombination events in a 6.4-Mb region covered by 68 HBMs and 21 SSR markers surrounding the *Ver1* locus on chromosome 16.

Supplementary Table S6. Analysis of possible target genes extracted from the veraison-specific DEGs.

Supplementary Table S7. Compilation of recently published studies on bi-parental populations or subpopulations compared to our genotyping and mapping approach.

Supplementary Information S1. Detailed *Vitis* DNA extraction protocol.
