# Supplementary material for: Genotyping-by-sequencing-based high-resolution mapping reveals a single candidate gene for the grapevine veraison locus Ver1
Source: Plant Physiol. 2024 May 14;196(1):244–60. doi: 10.1093/plphys/kiae272 (PMC11376399; doi:10.1093/plphys/kiae272)
Supplement: kiae272_Supplementary_Data [file kiae272_supplementary_data.zip › Supplementary_Data_23_04_2024.pdf]

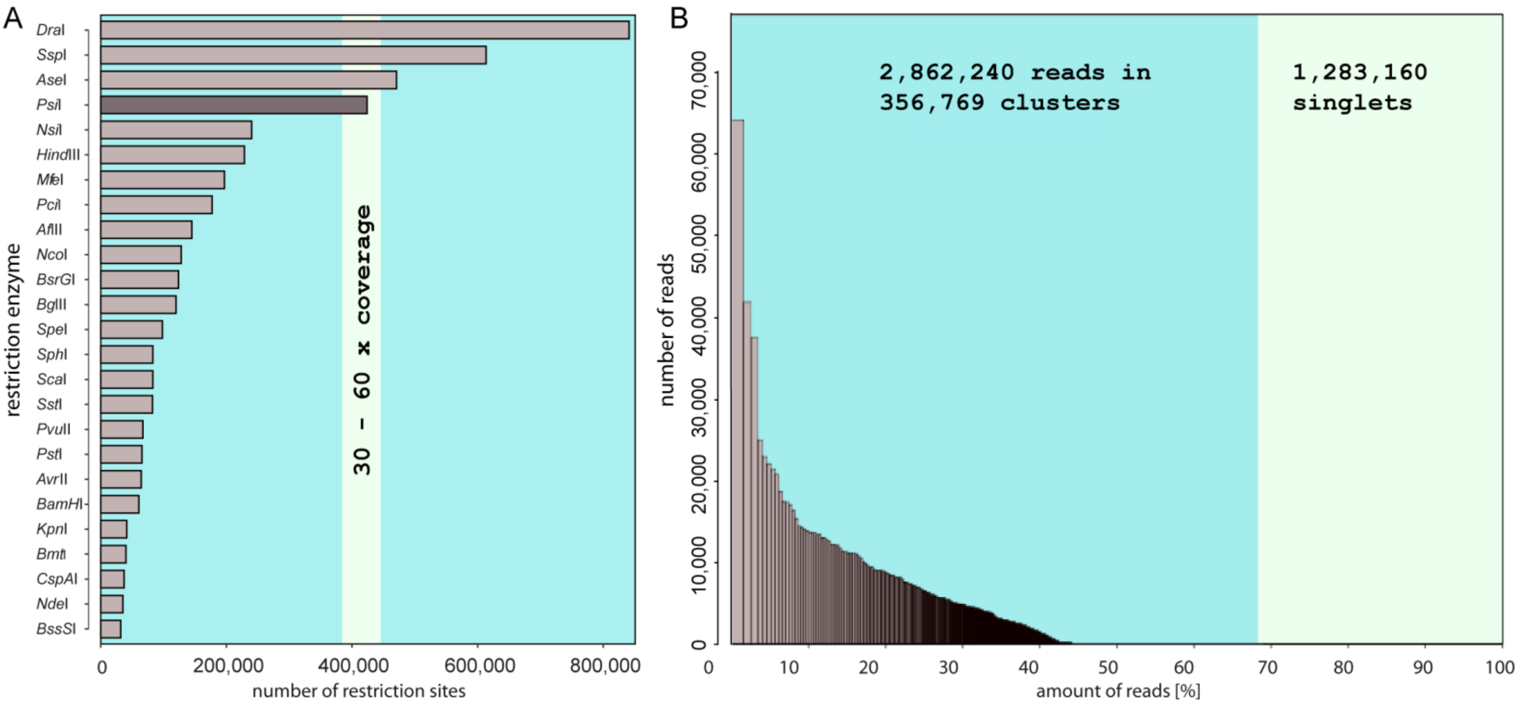

Supplementary Figure S1. ***In silico* tests (A) and genotyping-by-sequencing strategy design (B).** (A) 25 restriction enzymes were tested in an *in silico* digestion using the grapevine reference assembly (haploid, 486 Mb, Canaguier et al., 2017). (B) Barplot of the repeat clusters (turquoise) and singlet clusters (light green) uncovered by RepeatExplorer (Novak et al. 2013) for the Illumina raw data from SRR863595, https://www.ebi.ac.uk/ena/browser/view/SRR8635.

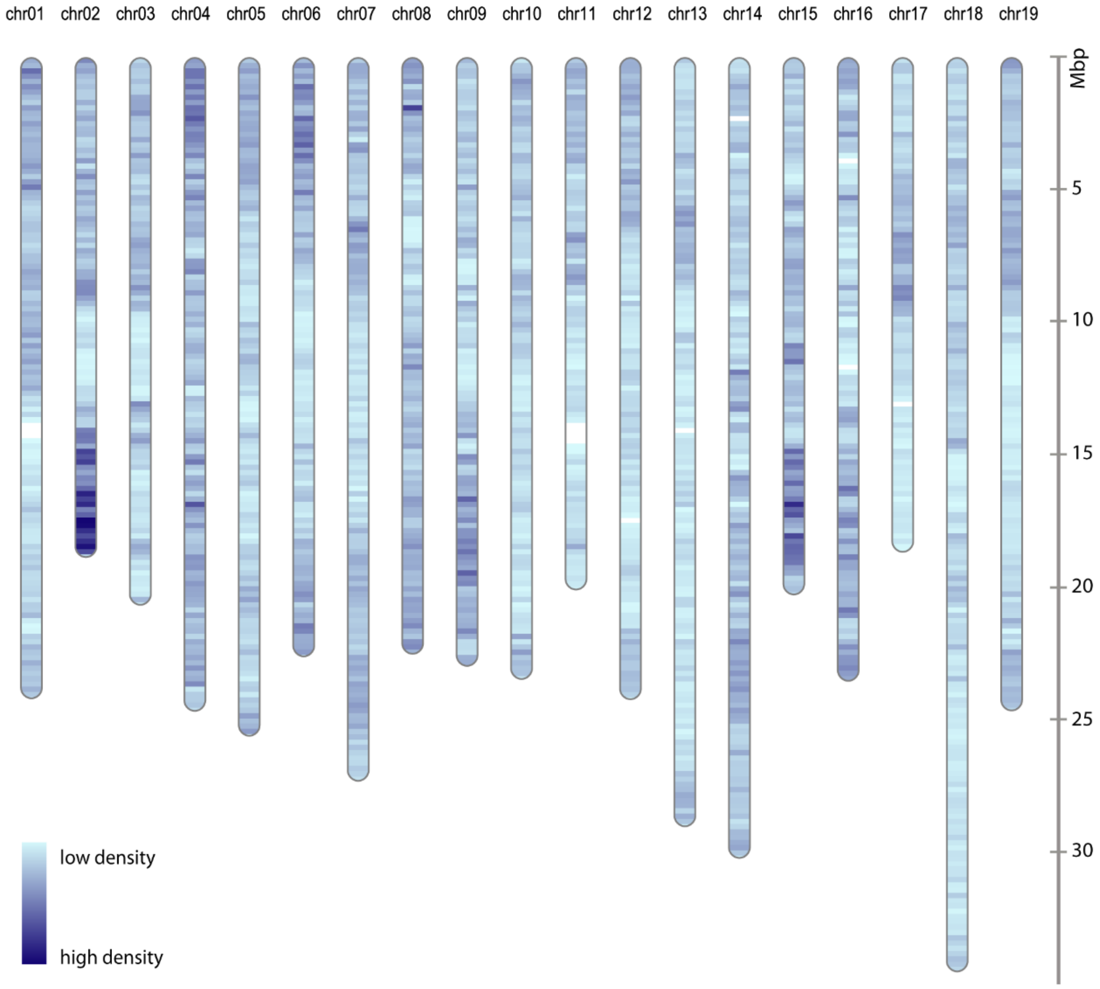

Supplementary Figure S2. Physical map of identified monomorphic loci mapped to the reference genome PN40024_12x v2 (Canaguier et al., 2017). Prominent regions with a high density of monomorphic loci were recorded for chromosomes 2, 4, 5 and 15. Mbp – Mega base pairs, chr – chromosome.

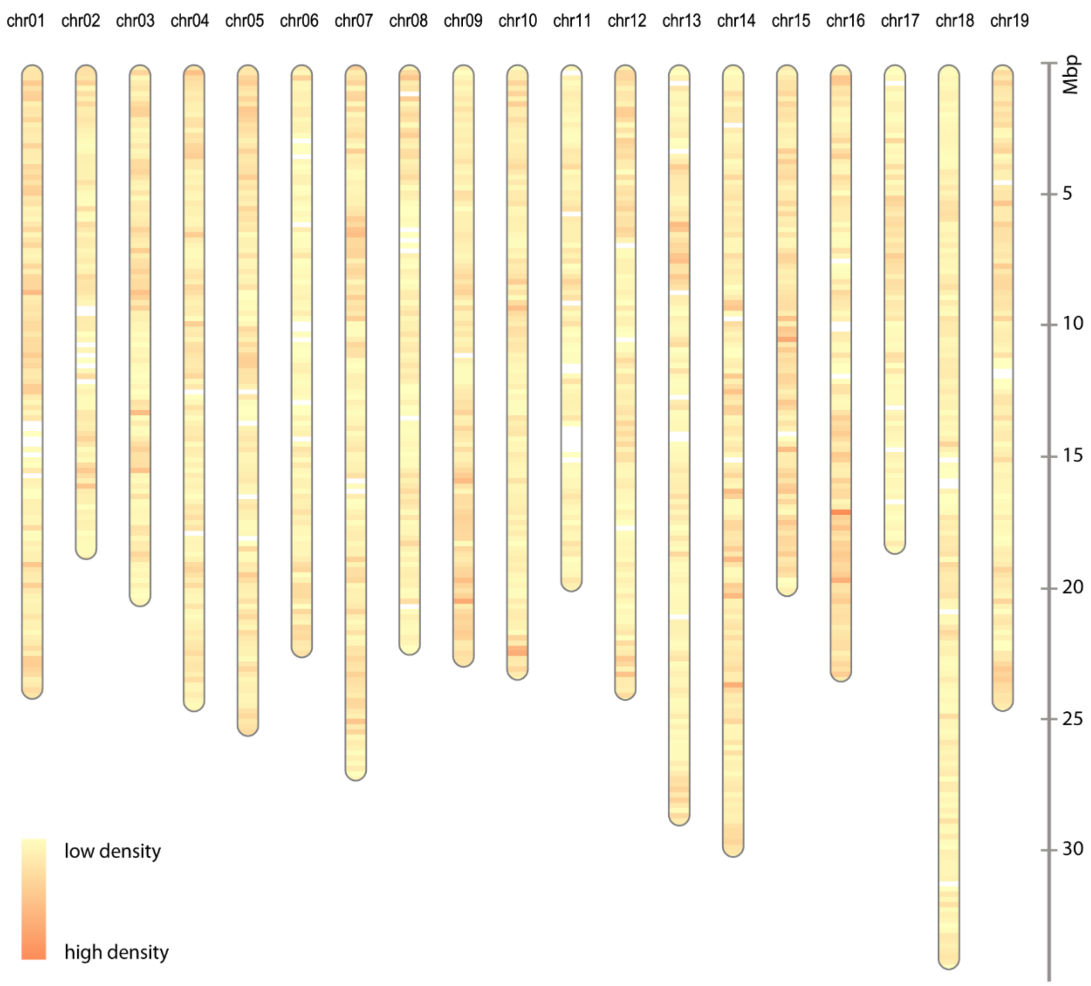

Supplementary Figure S3. Physical map of identified insertions/deletions (InDels) mapped to the reference genome PN40024_12×v2 (Canaguier et al., 2017). Of the 41,167 loci with 64,104 InDels, 18,136 were mapped. Mbp – Mega base pairs, chr – chromosome.

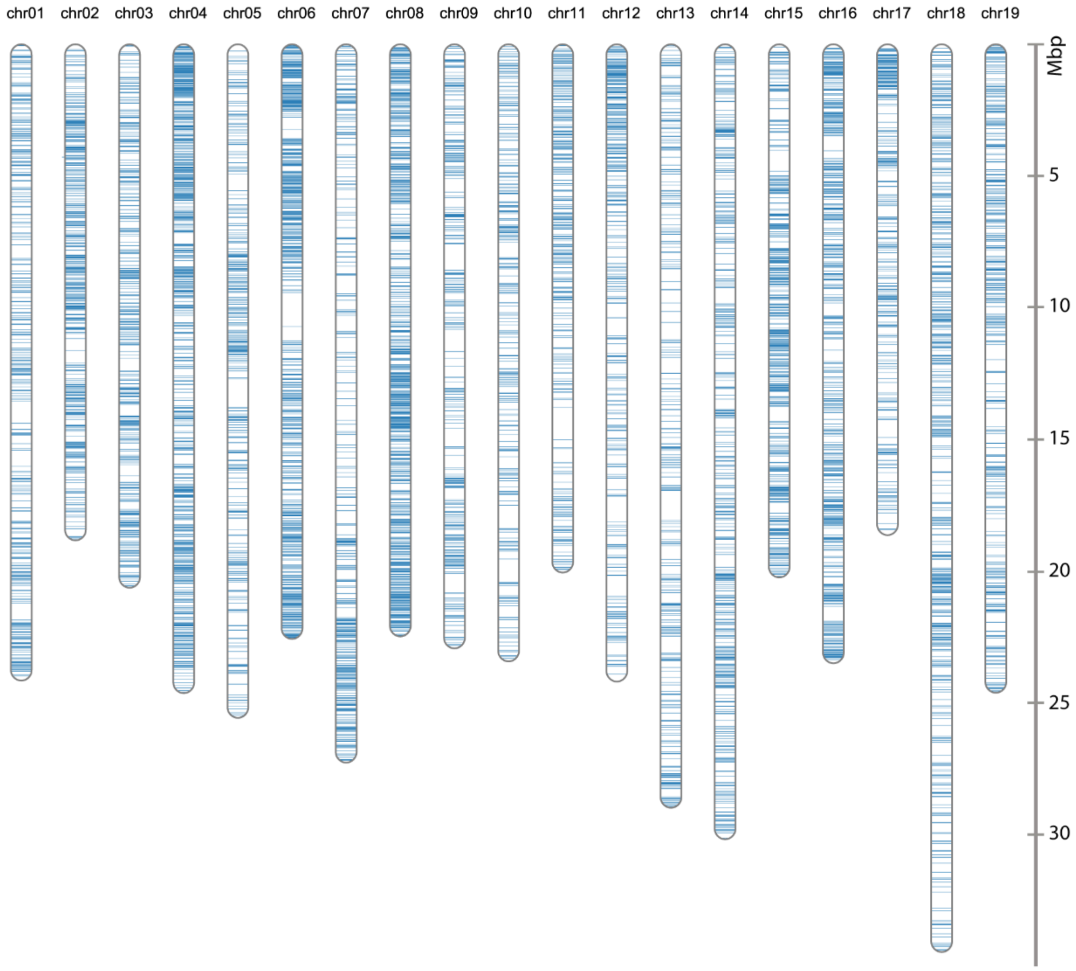

Supplementary Figure S4. Physical map of all loci with two possible alleles (2-allelic) that are shared by 85% of the F_1 progeny. Of all 2-allelic loci, 94.4% mapped to the reference genome PN40024_12×v2 (Canaguier et al., 2017). Mbp – Mega base pairs, chr – chromosome.

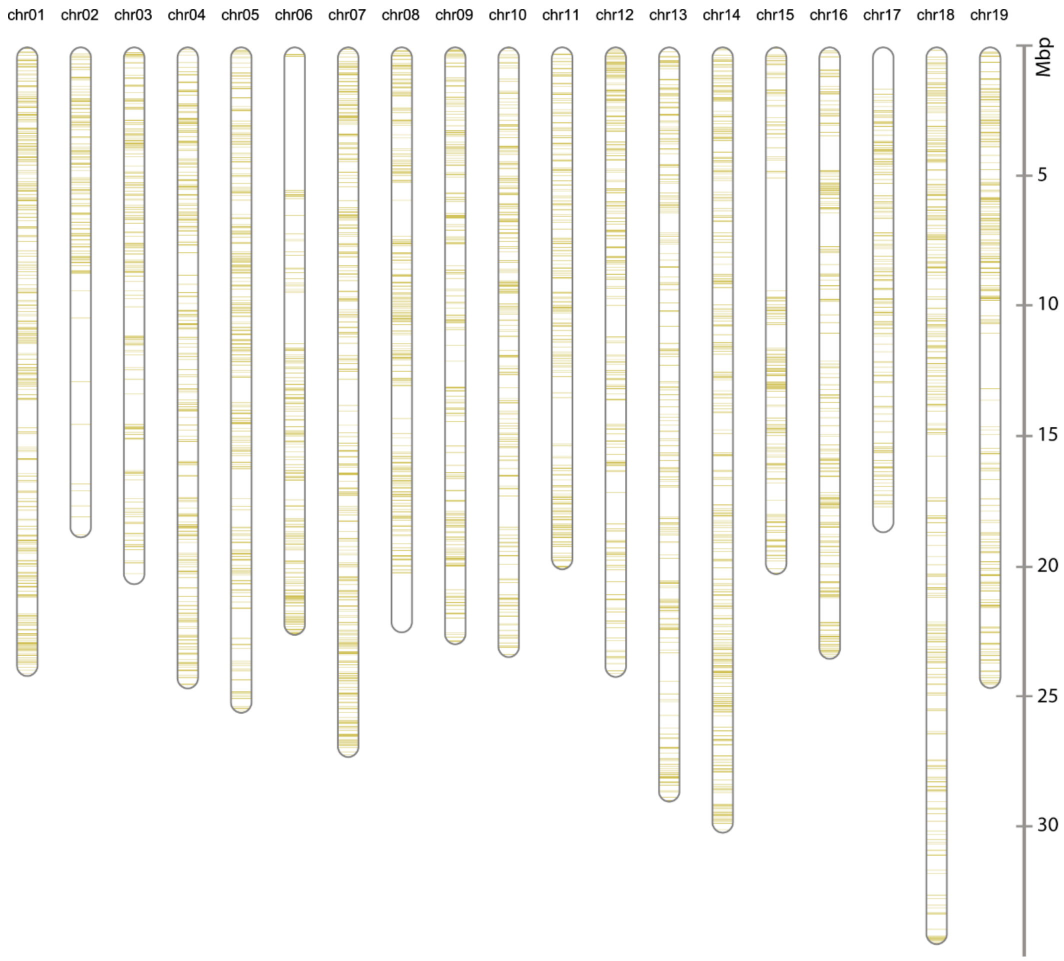

Supplementary Figure S5. Physical map of all loci with three possible alleles (3-allelic) that are shared by 85% of the F1 progeny. Of all 3-allelic loci, 96.5% mapped to the reference genome PN40024_12×v2 (Canaguier et al., 2017). Mbp – Mega base pairs, chr – chromosome.

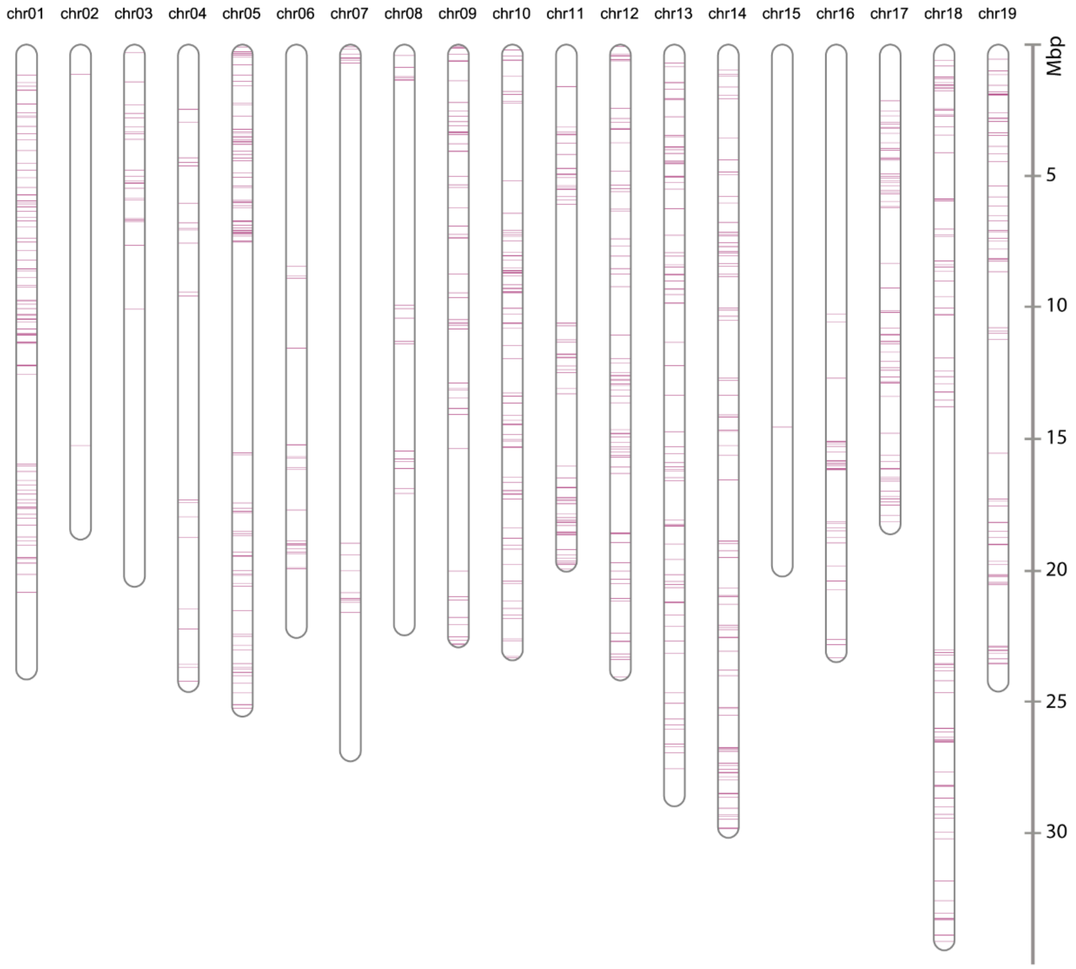

Supplementary Figure S6. Physical map of all loci with four possible alleles (4-allelic) that are shared by 85% of the F1 progeny. Of all 4-allelic loci, 98.3% mapped to the reference genome PN40024_12×v2 (Canaguier et al., 2017). Mbp – Mega base pairs, chr – chromosome.

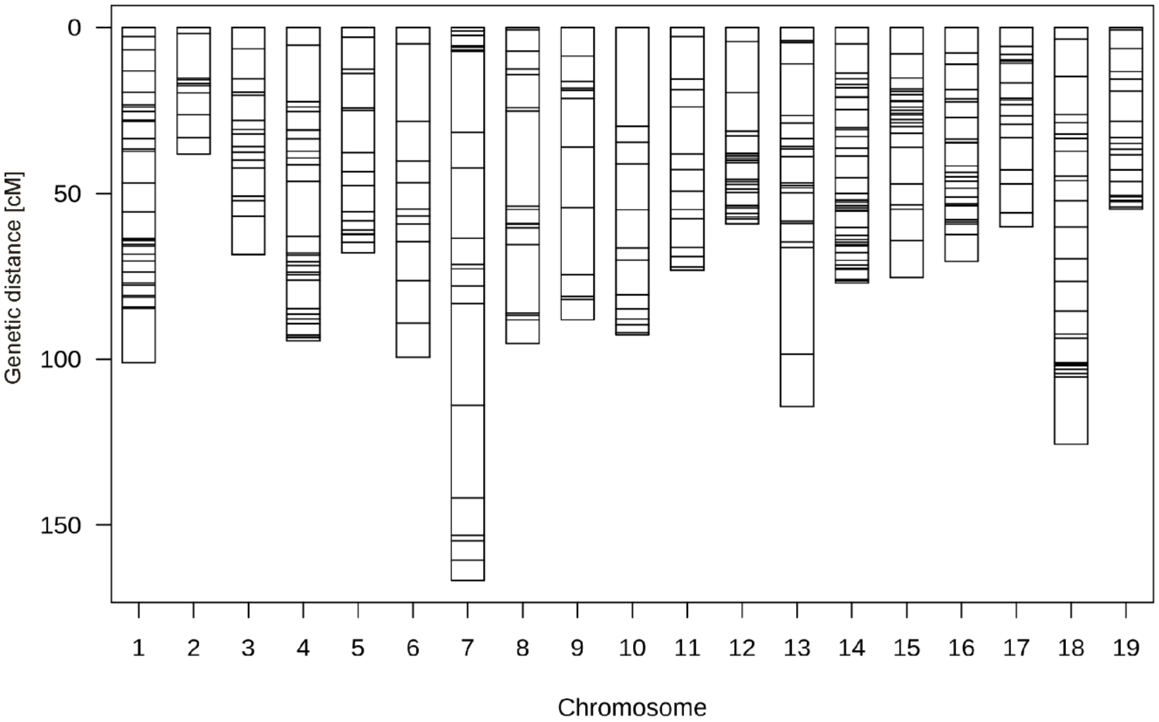

Supplementary Figure S7. Integrated genetic map based on SSR markers of the F1 population from the 'Calardis Musqué' × 'Villard blanc' cross. Linkage group numbers and orientation are based on the reference genome PN40024_12×v2 (Canaguier et al., 2017). SSR – Simple Sequence Repeat.

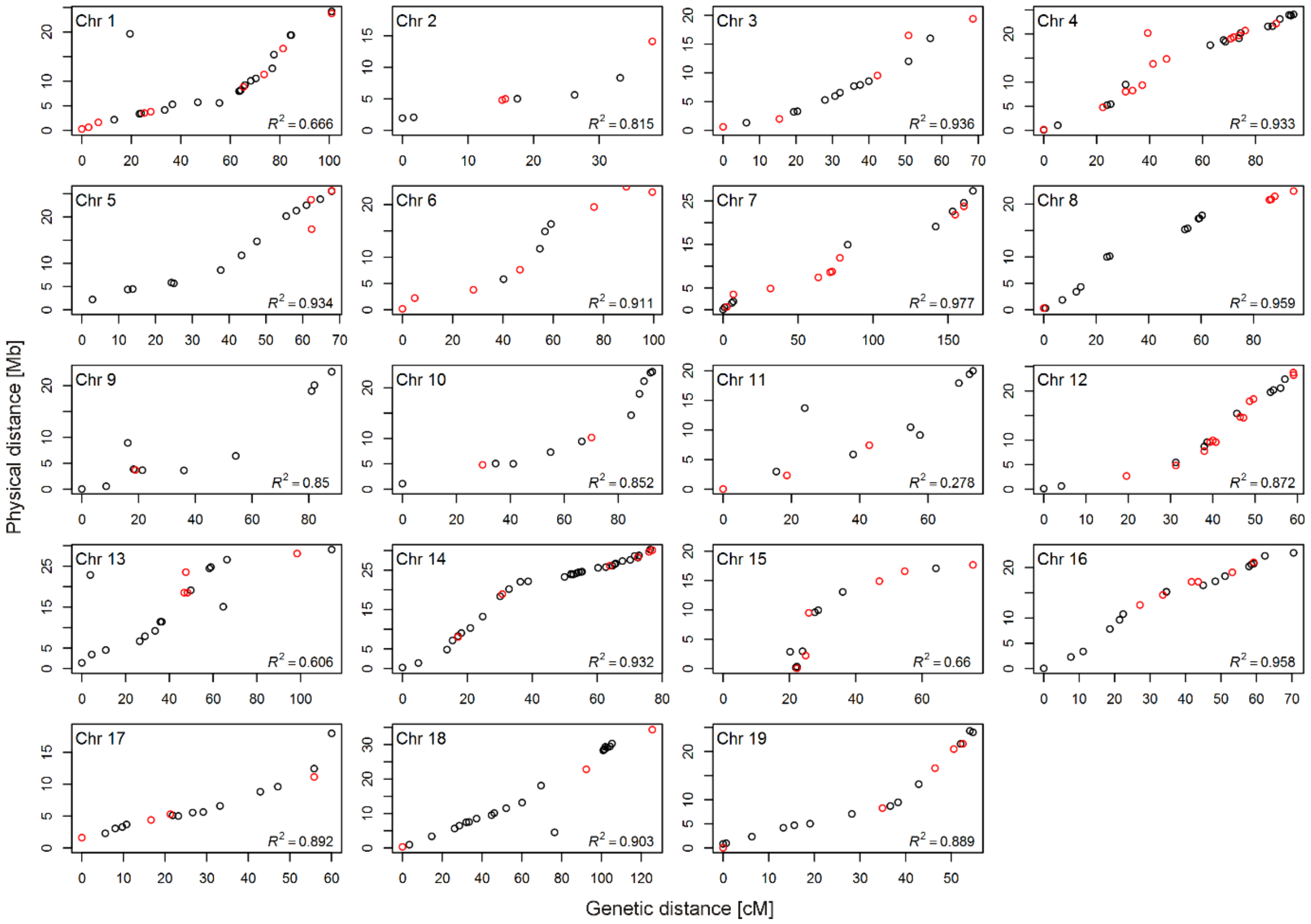

Supplementary Figure S8. Marey maps showing the coincidence between physical and genetic positions of the SSR map. Fully informative markers are indicated in black circles and partly informative markers in red circles. Chr – chromosome, SSR – Simple Sequence Repeat, Mb – megabase, cM – centimorgan.

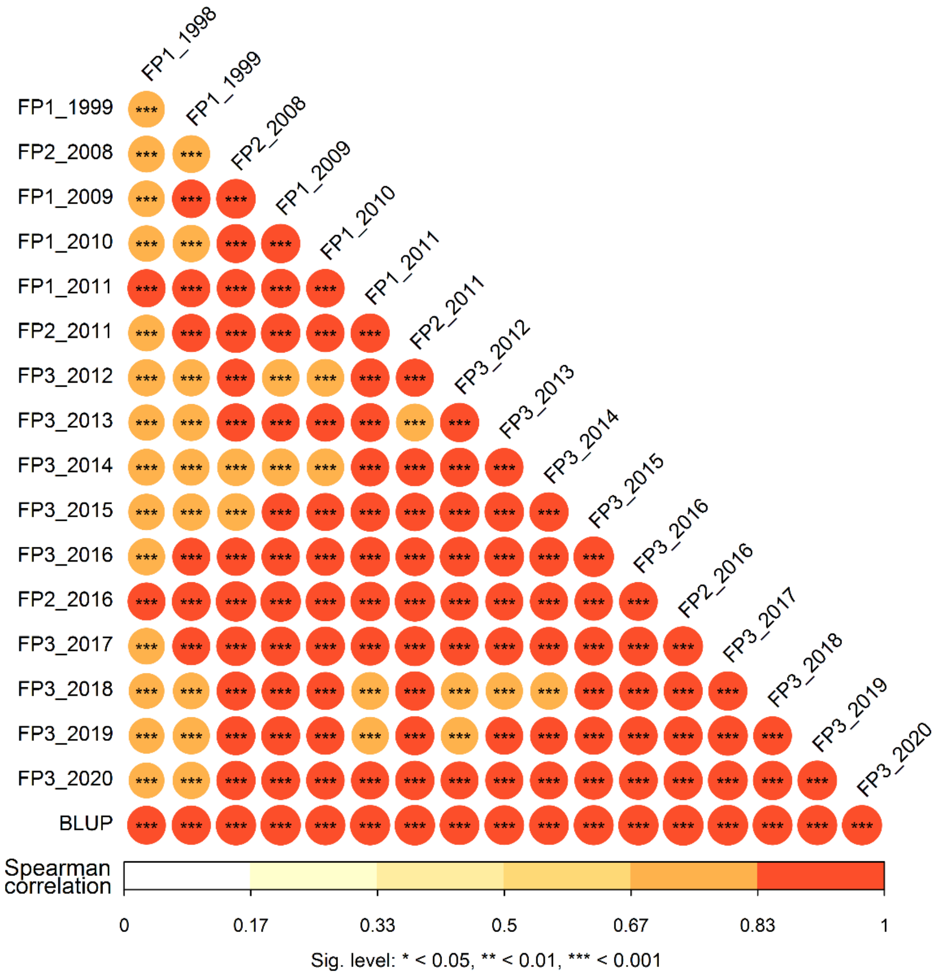

Supplementary Figure S9. Spearman correlation matrix between single veraison datasets. Color illustrates the correlation between pairs; asterisks the significance level. FP – field plot, BLUP – Best Linear Unbiased Prediction.

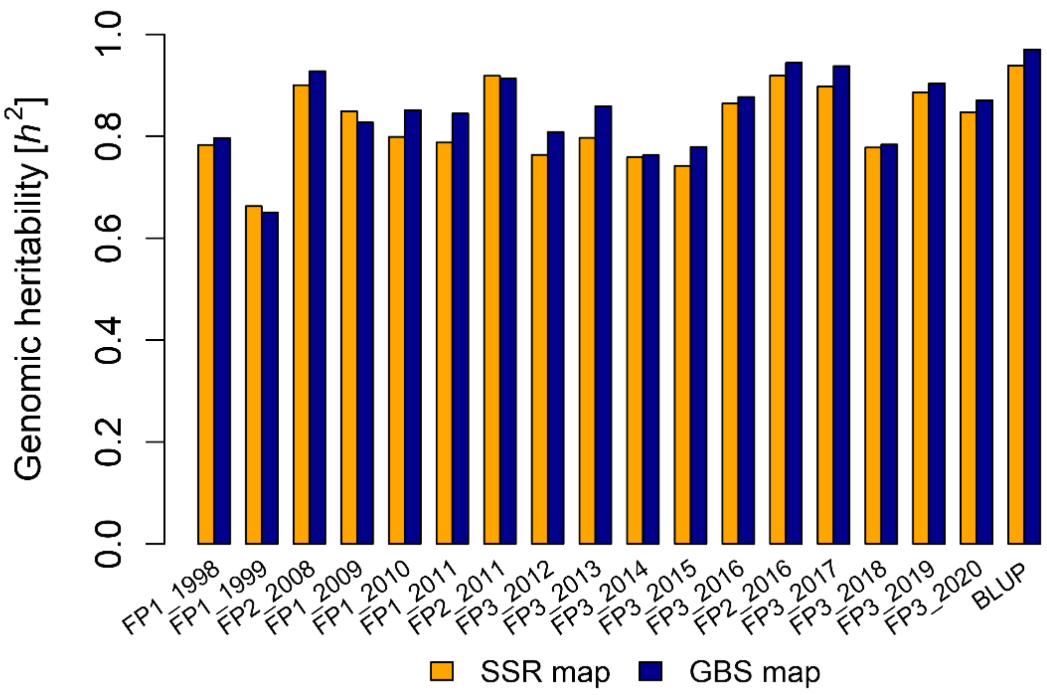

Supplementary Figure S10. Genomic heritability estimates (h2) of the veraison trait in single-year datasets using genomic relationships between individuals for each genetic map. FP – field plot, BLUP – Best Linear Unbiased Prediction, SSR – Simple Sequence Repeat, GBS – Genotyping-by-sequencing.

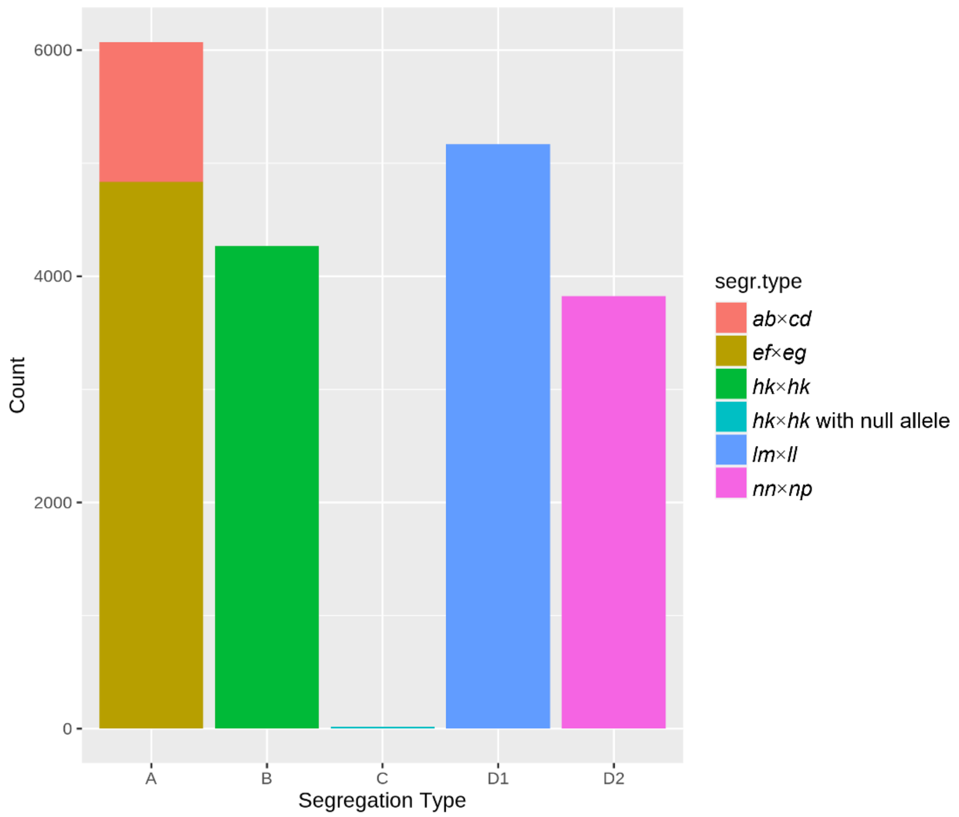

Supplementary Figure S11. Distribution of segregation types in the LSME approach for the 19,351 markers.

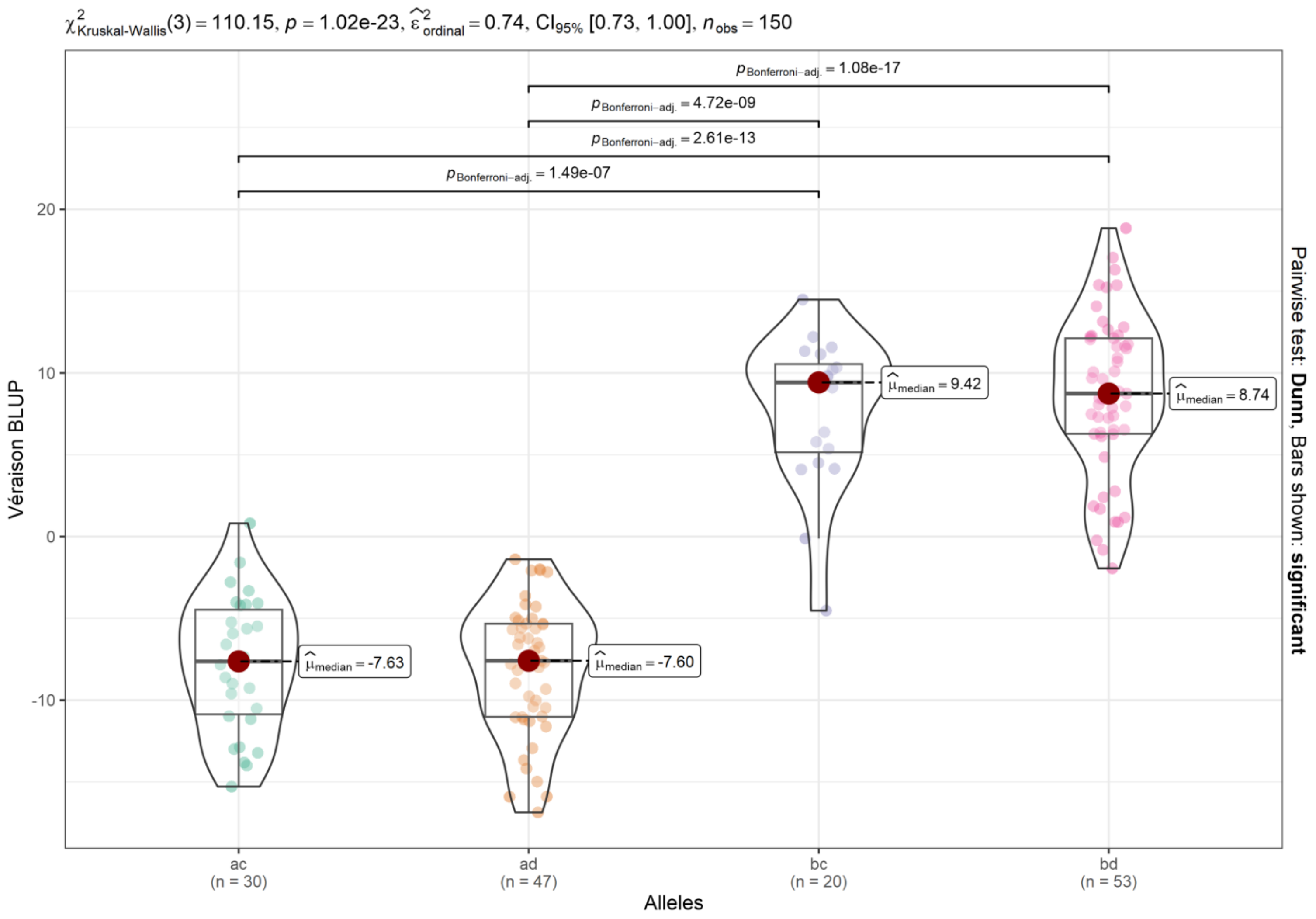

Supplementary Figure S12. Allelic effects on the Best Linear Unbiased Prediction (BLUP) of the veraison onset according to the genotypic classes "*ac*" (light green), "*ad*" (light orange), "*bc*" (light purple) and "*bd*" (light pink) at the marker UDV-052 showing the highest LOD value. Colored dots – single observations, center line with red dot – median, gray box (box plot) – interquartile range, straight gray lines (whiskers) – 1.5x interquartile range, outer lines (violin plot) – kernel density estimation, BLUP – Best Linear Unbiased Prediction.

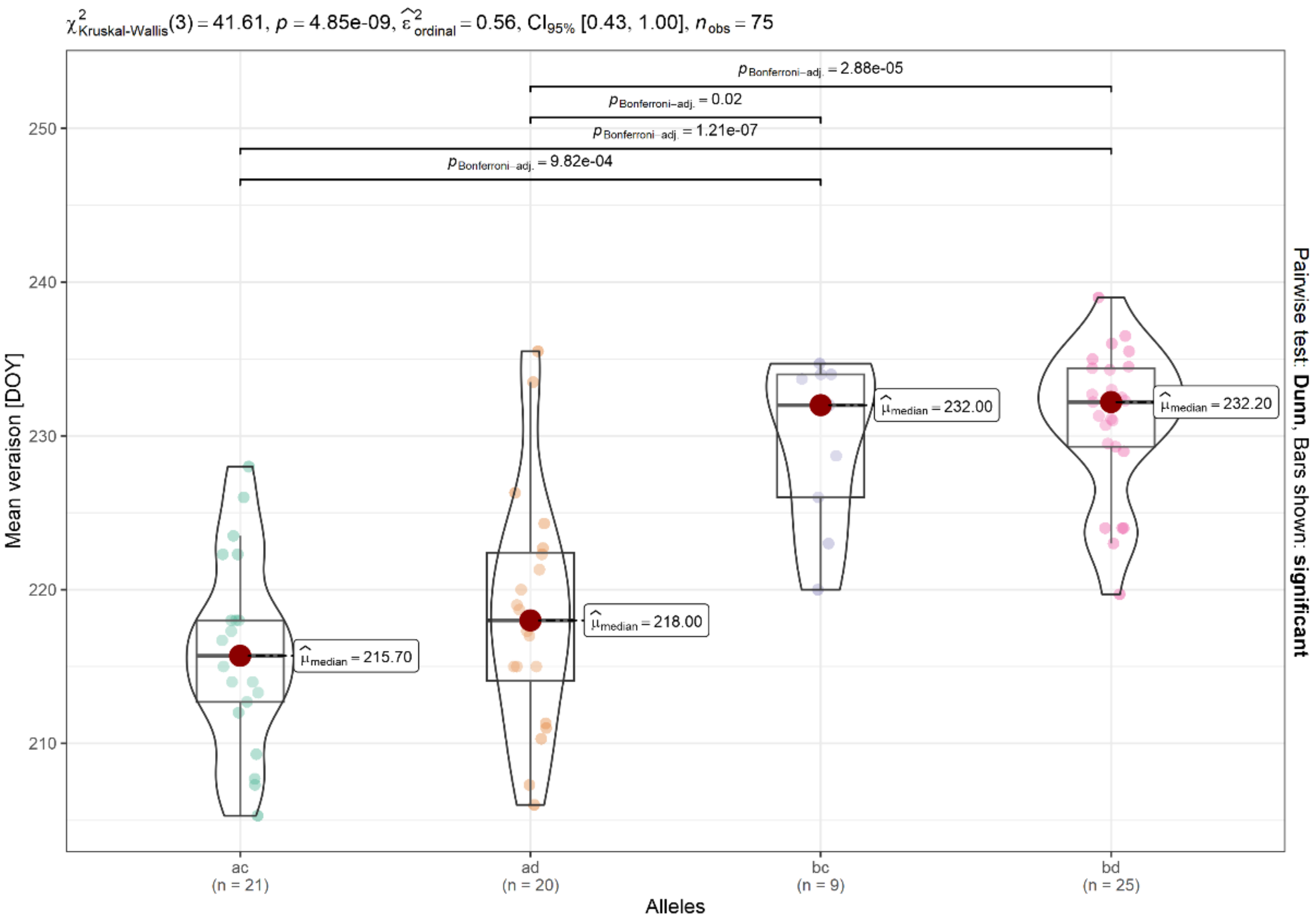

Supplementary Figure S13. Distribution of the mean veraison date of *Ver1* recombinant genotypes grouped by the genomic state of correlated marker GF16-75 with significant differentiation between maternal alleles *a* (light green, light orange) and *b* (light purple, light pink). Colored dots – single observations, centre line with red dot – median, gray box (box plot) – interquartile range, straight gray lines (whiskers) – 1.5x interquartile range, outer lines (violin plot) – kernel density estimation, DOY – day of year.

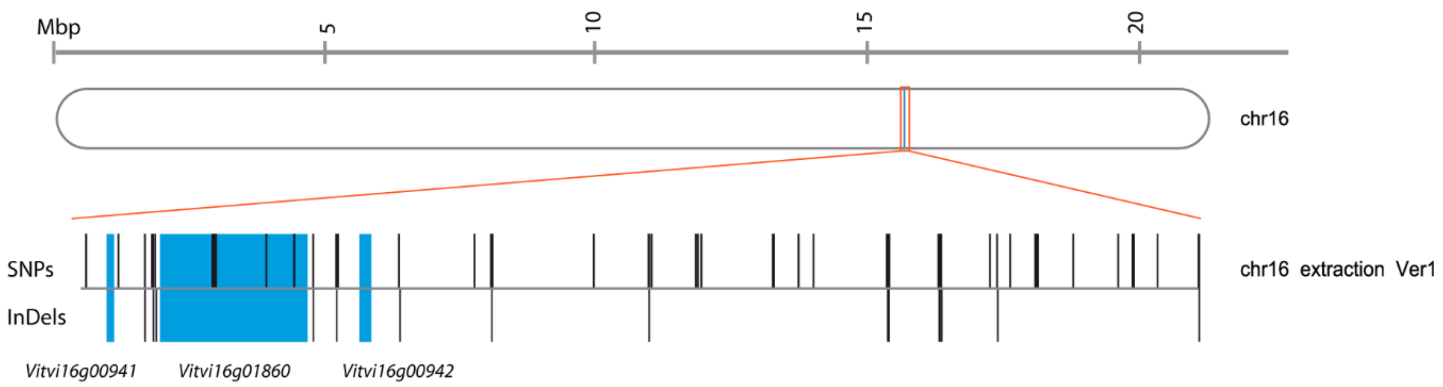

Supplementary Figure S14. Chromosome 16 with the 112 kb veraison locus (*Ver1*, orange box, above). Zoomed in region (below) shows the distribution of SNPs and InDels as well as positions of major candidate genes in the corresponding region (blue). Mbp – Mega base pairs, chr – chromosome, SNP – Single-nucleotide polymorphism, InDel – insertion-deletion polymorphism.

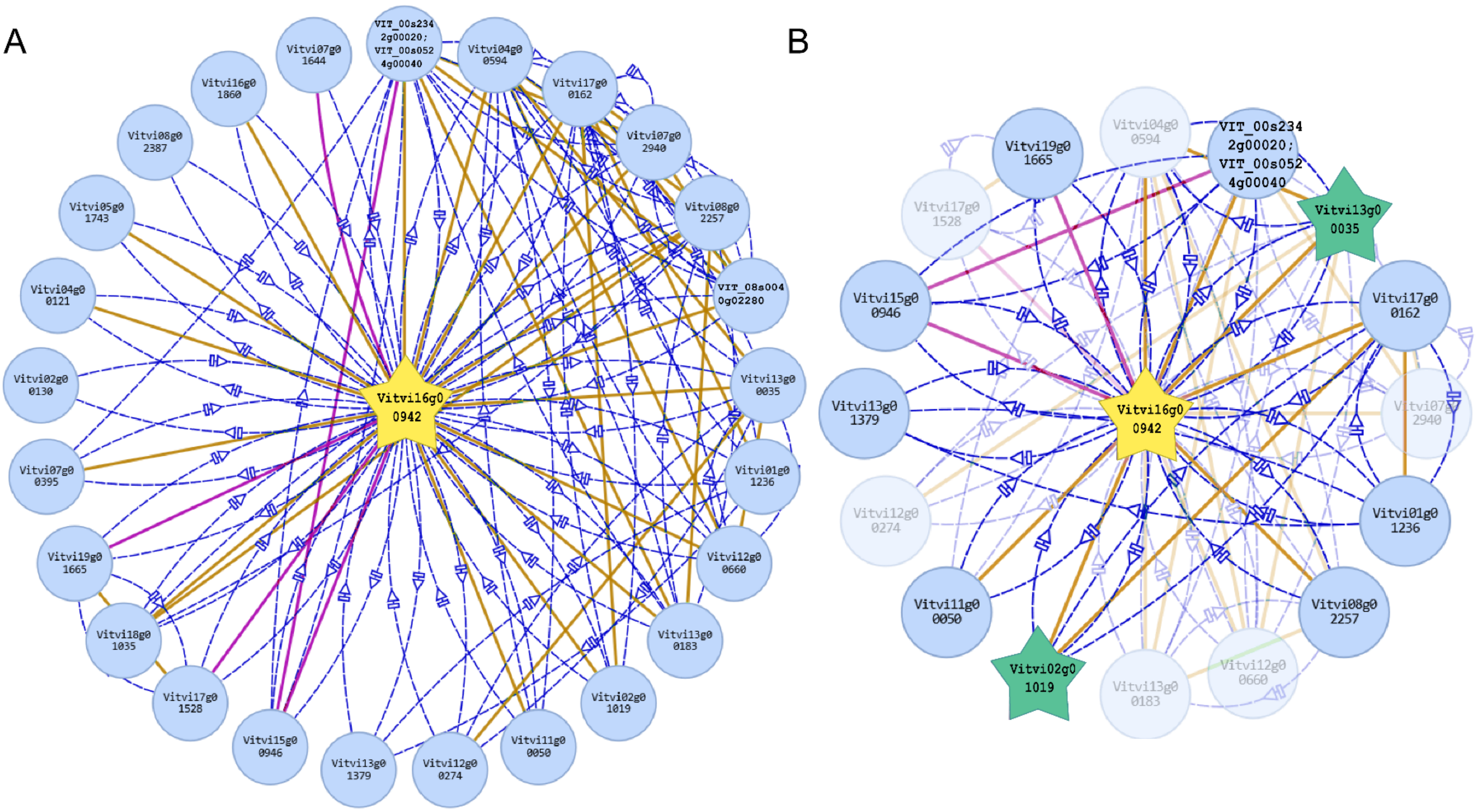

Supplementary Figure S15. OneGenE networks of our candidate gene *VvERF027* (Vitvi16g00942). (A) Expanded gene network with *VvERF027* as seed gene in the centre (yellow star). (B) Reduced OneGenE network of Vitvi16g00942 showing only genes shared with the 388 DEGs of Theine et al. (2021). Transparent circles display further directly associated genes. Mint green stars mark genes that contain the GCC box in their 2000 bp promoter region. Blue lines – OneGene relative frequency causal association value 0.95–1.00, arrows on lines – direction of association, ochre-colored lines – Pearson correlation value 0.60–1.00, magenta-colored lines – Pearson correlation value -1.00– -0.60, light-blue circles – standard representation of network genes.

Supplementary Information S1. Detailed *Vitis* DNA extraction protocol.

Twenty mg of dried leaf material was placed in a 2 ml microcentrifuge tube containing two 3 mm Tungsten carbide beads (Qiagen) and one spatula tip of sea sand (Supelco®). The tissue was homogenized by shaking twice for 30 s at a frequency of 25 Hz using a TissueLyser II (Qiagen). After centrifugation for 5 s, 1000 µl warm (65°C) CTAB extraction buffer (100 mM Tris-HCl, pH 8.0; 1.4 M NaCl; 20 mM EDTA; 2% PVP, 4% CTAB; 0.3% 2-Mercaptoethanol) was added to the leaf powder. The lysate was incubated at 65°C for 30 min, followed by 30 min shaking in a HulaMixer™ (360° vertical rotation) and 5 min centrifugation at 5,000 x g at room temperature (RT). The supernatant was transferred to a new 2 ml microcentrifuge tube and mixed with an equal volume of Chloroform:Isoamylalcohol (24:1). After gentle shaking for 5 min, the mixture was centrifuged for 10 min at 20,000 x g at RT. The upper organic phase was transferred to a new tube and incubated with 0.02 mg of RNase A (ThermoFisherScientific, 10 mg/ml) at 37°C for further 60 min. An equal volume of Chloroform:Isoamylalcohol (24:1) was added to the extract, which was again shaken for 5 min and centrifuged for 10 min at 20,000 x g at RT. The upper organic phase was transferred to a new 1.5 ml microcentrifuge tube and mixed with 0.7 volume of 2-propanol for precipitation. After incubation for 10 min at RT, the mixture was centrifuged for 30 min at 20,000 x g at 4°C. The resulting pellet was then washed twice with 70% ethanol, dried at 60°C and finally dissolved in 80 µl HPLC grade water (AppliChem GmbH) at 60°C.

Supplementary Table S7. Compilation of recently published studies on bi-parental populations or subpopulations compared to our genotyping and mapping approach.

	present study	*Vervalle et al. (2022)*	*Shi et al. (2022)*	*Possamai et al. (2021)*	*Duchêne et al. (2020)*	*Su et al. (2020)*	*Jiang et al. (2020)*	*Fu et al. (2020)*	*Tello et al. (2019)*	*Sapkota et al. (2019)*	*Zhu et al. (2018)*	*Hyma et al. (2015)*
**population design**	bi-parental, 147 geno-types	multi-parental, ~ 140 geno-types/population	bi-parental, 92 hybrid geno-types	bi-parental, 184 geno-types	bi-parental, 120 geno-types	bi-parental, 181 hybrid geno-types	bi-parental, 105 geno-types	bi-parental, 91 hybrid geno-types	multi-parental, ~70 geno-types/subpopulation	bi-parental, 182 geno-types	bi-parental, 176 geno-types	5 half sib, interspecific F1 families
**genotyping approach**	GBS, *Psi*I (not me-sensitive)	Illumina Vitis18K SNP	whole-genome resequencing	GBS, *Ape*KI	SSR + Illumina Grape20K Infinium ® BeadChip	RAD-Seq	whole-genome sequencing	GBS, *Mse*I (not me-sensitive) and *Hae*II (me-sensitive)	GBS, *Ape*KI	GBS, *Ape*KI	RAD-Seq	GBS, *Ape*KI
**no of loci/ population**	495,757	~18,000	?	695,985	18,071	~1,000,000?	?	?	~215,240 (2,152,397 for all 10 crosses)	43,971	~ 1,426,967	852,885 - 1,219,257 tags
**pipeline design**	*de novo* clustering	alignment to the *Vitis vinifera* PN40024 reference genome	alignment to the *Vitis vinifera* PN40024 reference genome	alignment to the *Vitis vinifera* PN40024 reference genome	alignment to the *Vitis vinifera* PN40024 reference genome	alignment to the *Vitis vinifera* PN40024 reference genome	alignment to the *Vitis vinifera* PN40024 reference genome	alignment to the *Vitis vinifera* PN40024 reference genome	alignment to the *Vitis vinifera* PN40024 reference genome	alignment to the *Vitis vinifera* PN40024 reference genome	alignment to the *Vitis vinifera* PN40024 reference genome	*de novo* linkage group (LG) formation
**SNP number/ population**	561,795 ~4 SNPs/ locus	=no of loci	3,443,756	=no of loci	= no of loci	?	27,695	344,782	= no of loci	= no of loci	?	300,773 - 449,840
**no of indels/ population**	64,104	-	-	-	-	-	-	-	-	-	> 4832	-

**no of markers/ population**	20,410 haplotype - based markers	6326-7175	1351	2291 - 2627	6837 (incl. 2021 hk × hk discarded)	25,917 SNP markers	1662 bin markers (lm × ll, nn × np, hk × hk)	5603 (lm × ll, nn × np, hk × hk)	6170 – 23,470	3825	65,229	5343-11,080
**no of full informative markers/ population (ab × cd, eg × ef)**	5,936	0	674 (from initial 210,873 SNPs)	?	0	89	0	1578 (from initial 344,782)	667-2147	255 (of 2072 applied markers)	64 (incl. InDel markers)	?
**map size/ population (cM)**	1152.29 (integrated map)	1259 (integrated map)	860.46	1205 - 1315	1413.1	1780 (integrated map)	1463.38 (integrated map)	1898.09 (integrated map)	1065.2 to 1367.3	2203.5 (integrated map)	3014.46 (integrated map)	1125 - 1696
**average marker distance (cM)**	0.51	0.60	0.65	0.50-0.52	1.5	0.41	0.88	0.35	1.8-3.7	0.8-1.9	0.05	0.24-1.4

References

Duchêne É, Dumas V, Butterlin G, Jaegli N, Rustenholz C, Chauveau A, Bérard A, Le Paslier MC, Gaillard I, Merdinoglu D (2020) Genetic variations of acidity in grape berries are controlled by the interplay between organic acids and potassium. Theor Appl Genet 133: 993–1008

Fu P, Tian Q, Lai G, Li R, Song S, Lu J (2019) Cgr1, a ripe rot resistance QTL in *Vitis amurensis* ‘Shuang Hong’ grapevine. Hortic Res 6: 1–9

Hyma KE, Barba P, Wang M, Londo JP, Acharya CB, Mitchell SE, Sun Q, Reisch B, Cadle-Davidson L (2015) Heterozygous Mapping Strategy (HetMappS) for High Resolution Genotyping-By-Sequencing Markers: A Case Study in Grapevine. PLOS ONE 10: e0134880

Jiang J, Fan X, Zhang Y, Tang X, Li X, Liu C, Zhang Z (2020) Construction of a High-Density Genetic Map and Mapping of Firmness in Grapes (*Vitis vinifera* L.) Based on Whole-Genome Resequencing. International Journal of Molecular Sciences 21: 797

Possamai T, Wiedemann-Merdinoglu S, Merdinoglu D, Migliaro D, De Mori G, Cipriani G, Velasco R, Testolin R (2021) Construction of a high-density genetic map and detection of a major QTL of resistance to powdery mildew (*Erysiphe necator* Sch.) in Caucasian grapes (*Vitis vinifera* L.). BMC Plant Biology 21: 528

Sapkota S, Chen L-L, Yang S, Hyma KE, Cadle-Davidson L, Hwang C-F (2019) Construction of a high-density linkage map and QTL detection of downy mildew resistance in *Vitis aestivalis*-derived 'Norton.' Theor Appl Genet 132: 137–147

Shi G, Sun D, Wang Z, Liu X, Guo J, Zhang S, Zhao Y, Ai J (2022) Construction of a resequencing-based high-density genetic map for grape using an interspecific population (*Vitis amurensis* × *Vitis vinifera*). Hortic Environ Biotechnol 63: 489–497

Su K, Xing H, Guo Y, Zhao F, Liu Z, Li K, Li Y, Guo X (2020) High-density genetic linkage map construction and cane cold hardiness QTL mapping for *Vitis* based on restriction site-associated DNA sequencing. BMC Genomics 21: 419

Tello J, Roux C, Chouiki H, Laucou V, Sarah G, Weber A, Santoni S, Flutre T, Pons T, This P, et al (2019) A novel high-density grapevine (*Vitis vinifera* L.) integrated linkage map using GBS in a half-diallel population. Theoretical and Applied Genetics. doi: 10.1007/s00122-019-03351-y

Vervalle JA, Costantini L, Lorenzi S, Pindo M, Mora R, Bolognesi G, Marini M, Lashbrooke JG, Tobutt KR, Vivier MA, et al (2022) A high-density integrated map for grapevine based on three mapping populations genotyped by the *Vitis*18K SNP chip. Theor Appl Genet 135: 4371–4390

Zhu J, Guo Y, Su K, Liu Z, Ren Z, Li K, Guo X (2018) Construction of a highly saturated Genetic Map for *Vitis* by Next-generation Restriction Site-associated DNA Sequencing. BMC Plant Biol 18: 347
